# Prioritizing surveillance of Nipah virus in India

**DOI:** 10.1371/journal.pntd.0007393

**Published:** 2019-06-27

**Authors:** Raina K. Plowright, Daniel J. Becker, Daniel E. Crowley, Alex D. Washburne, Tao Huang, P. O. Nameer, Emily S. Gurley, Barbara A. Han

**Affiliations:** 1 Department of Microbiology and Immunology, Montana State University, Bozeman, MT, United States of America; 2 Center for the Ecology of Infectious Disease, University of Georgia, Athens, GA, United States of America; 3 Cary Institute of Ecosystem Studies, Millbrook, NY, United States of America; 4 Centre for Wildlife Studies, College of Forestry, Kerala Agricultural University KAU (PO), Thrissur, Kerala, India; 5 Department of Epidemiology, Johns Hopkins Bloomberg School of Public Health, Baltimore, Maryland, United States of America; Molecular Biology Unit (MBU), INDIA

## Abstract

The 2018 outbreak of Nipah virus in Kerala, India, highlights the need for global surveillance of henipaviruses in bats, which are the reservoir hosts for this and other viruses. Nipah virus, an emerging paramyxovirus in the genus *Henipavirus*, causes severe disease and stuttering chains of transmission in humans and is considered a potential pandemic threat. In May 2018, an outbreak of Nipah virus began in Kerala, > 1800 km from the sites of previous outbreaks in eastern India in 2001 and 2007. Twenty-three people were infected and 21 people died (16 deaths and 18 cases were laboratory confirmed). Initial surveillance focused on insectivorous bats (*Megaderma spasma*), whereas follow-up surveys within Kerala found evidence of Nipah virus in fruit bats (*Pteropus medius*). *P*. *medius* is the confirmed host in Bangladesh and is now a confirmed host in India. However, other bat species may also serve as reservoir hosts of henipaviruses. To inform surveillance of Nipah virus in bats, we reviewed and analyzed the published records of Nipah virus surveillance globally. We applied a trait-based machine learning approach to a subset of species that occur in Asia, Australia, and Oceana. In addition to seven species in Kerala that were previously identified as Nipah virus seropositive, we identified at least four bat species that, on the basis of trait similarity with known Nipah virus-seropositive species, have a relatively high likelihood of exposure to Nipah or Nipah-like viruses in India. These machine-learning approaches provide the first step in the sequence of studies required to assess the risk of Nipah virus spillover in India. Nipah virus surveillance not only within Kerala but also elsewhere in India would benefit from a research pipeline that included surveys of known and predicted reservoirs for serological evidence of past infection with Nipah virus (or cross reacting henipaviruses). Serosurveys should then be followed by longitudinal spatial and temporal studies to detect shedding and isolate virus from species with evidence of infection. Ecological studies will then be required to understand the dynamics governing prevalence and shedding in bats and the contacts that could pose a risk to public health.

## Author summary

Nipah virus is an emerging zoonotic virus that spills over from bats to humans causing severe disease and chains of transmission in humans. In May 2018, in Kerala, India, Nipah virus infected 23 people, killing 21. We reviewed and analyzed published records of Nipah virus surveillance in bats and identified eleven species that occur in India and have had evidence of henipavirus infection or exposure. However, almost all of these bat species were sampled outside of India. Using a trait-based machine learning approach, we identified at least four additional Indian bat species that are likely to have been exposed to Nipah virus or cross-reacting henipaviruses. We suggest surveillance of these species as well as studies on the ecological dynamics of Nipah virus and epidemiology of spillover transmission to humans. This work will help prioritize a research agenda for responding to the recent outbreak of Nipah virus in Kerala, India and elsewhere.

## Introduction

The henipaviruses, including Nipah virus and Hendra virus, are highly lethal, emerging, bat-borne viruses within the family *Paramyxoviridae* that infect humans directly or via domestic animals that function as bridging hosts [1, 2]. Previous Nipah virus outbreaks were reported in Malaysia in 1998 [3], in eastern India in 2001 and 2007 [4–6], in Bangladesh almost annually since 2001 [7], and in Kerala, India, in May 2018 [8, 9]. In Malaysia, transmission from bats to humans occurred through pigs as intermediate hosts. Pigs were putatively infected after consuming fruit that was partially consumed by *Pteropus vampyrus* bats [10]. In Bangladesh, transmission from bats to humans occurred through consumption of date palm sap contaminated by *P*. *medius* (formerly *P*. *giganeteus*) [11], and subsequent human-to-human transmission has been commonly observed [12]. Initial wildlife studies in response to the 2018 Kerala outbreak focused on insectivorous bats (*Megaderma spasma*) [13], whereas a later survey focused on *P*. *medius* and found that 19% (10/52) of the *P*. *medius* tested had at least one biological sample with evidence of Nipah virus RNA using real-time reverse transcription polymerase chain reaction (RT-PCR). Details such as whether the bats originated from one or more populations and which tissues or specimens were sampled have not been published [9]. The routes of transmission from bats to the index case in Kerala are also unknown [14]. However, a salient feature of the outbreak in Kerala was the superspreader, who, while he was cared for in hospital, infected most cases identified during the outbreak [15]. Henipaviruses such as Nipah virus warrant attention from the global health community because of their ability to spread from person-to-person, although our understanding of which strains are most transmissible among humans, and why, is poor.

Henipaviruses circulate in bats throughout Asia, Africa, Australia, and the Americas [16–19]. Current understanding of the dynamics of henipaviruses in bats is based on studies of Hendra virus in Australia [20–26]; Nipah virus in Thailand [27], Bangladesh [7], Malaysia [10, 28, 29], and Cambodia [30]; and uncharacterized African bat henipaviruses in Madagascar [31], West Africa [32, 33], and pancontinental Africa [34, 35]. Although henipaviruses are widely distributed geographically, most surveillance has been patchy in space and time, and it seems likely that henipaviruses occur in species that have not yet been identified as reservoirs [18].

An additional challenge to confirming henipavirus reservoirs and characterizing their dynamics is the generally low and variable prevalence in bats [20, 21, 36]. Intensive spatial and temporal sampling is necessary to overcome these challenges, and such studies have yet to be conducted in India. Importantly, surveillance for human infections and further epidemiologic investigation provides crucial context for understanding which reservoir species are epidemiologically important, when and where spillovers occur, and which viruses pose the greatest public health threat.

To provide guidance for sampling bats in India generally, and guidance for epidemiologic studies looking for animal exposures associated with Nipah virus spillovers in Kerala, we systematically searched the literature for records of studies of Nipah virus and henipaviruses in bat species known to occur in Asia, Australia, and Oceana. We collated all records of Nipah virus shedding from bats (PCR) and Nipah virus exposure in bats (serology that likely includes cross-reacting henipaviruses). We used generalized boosted regression of more-extensive data on bats in Asia, Australia, and Oceana to make trait-based predictions of likely henipavirus reservoirs near Kerala.

## Methods

### Detection of Nipah virus in bats

**Fig 1:**
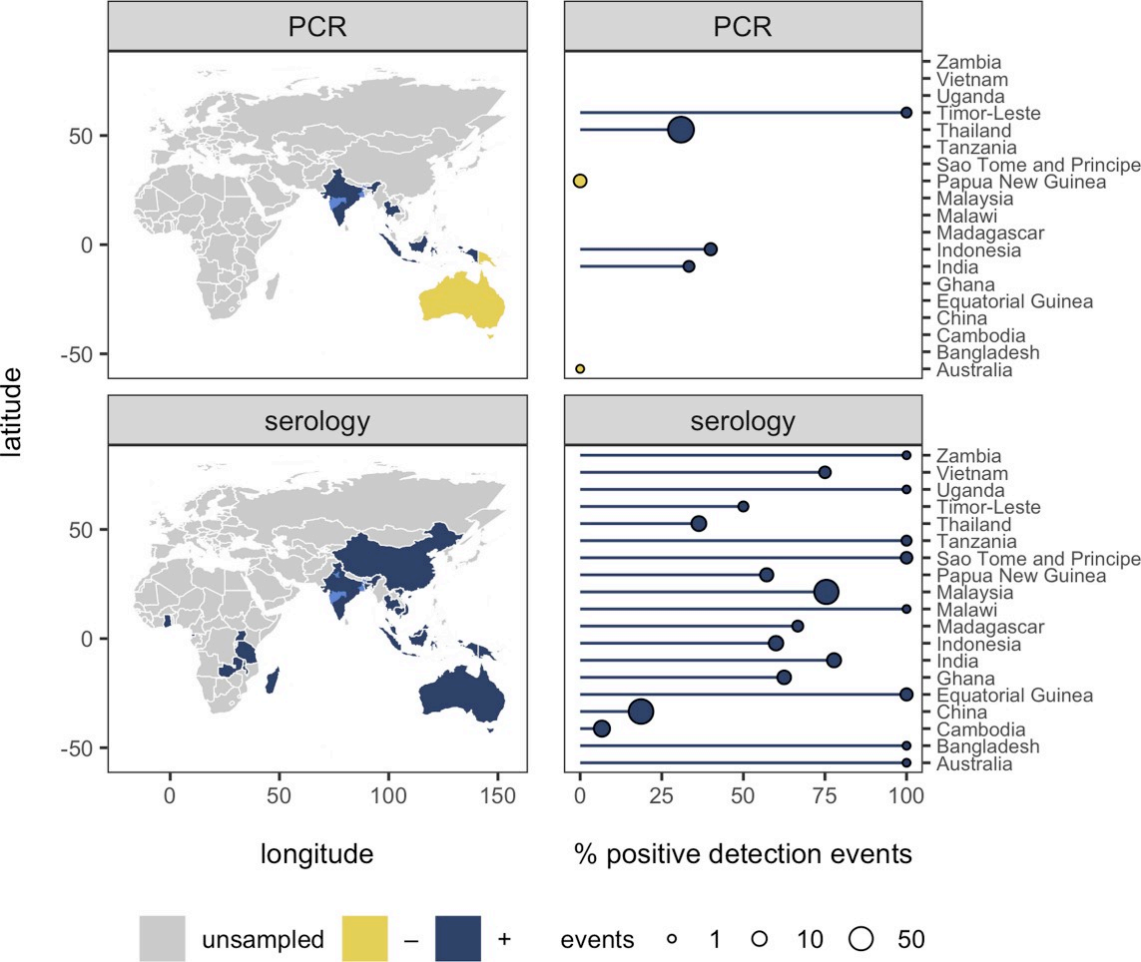
Global map of Nipah virus sampling effort and detections. The country-level distribution of whether bats have been sampled for Nipah virus and whether positive detections have occurred using PCR or serology (noting that serological detection likely includes cross-reactions with Nipah-like viruses). Light blue shading shows regions of India where bats have been found positive for Nipah virus (states of Haryana, Maharashtra, West Bengal); studies did not report exact sampling coordinates [40, 41]. Shapefiles were obtained from the *maps* and *mapdata* packages in R. The right panels show the number of sampling events and the percentage of positive detections by PCR or serology in each country.

**Fig 2:**
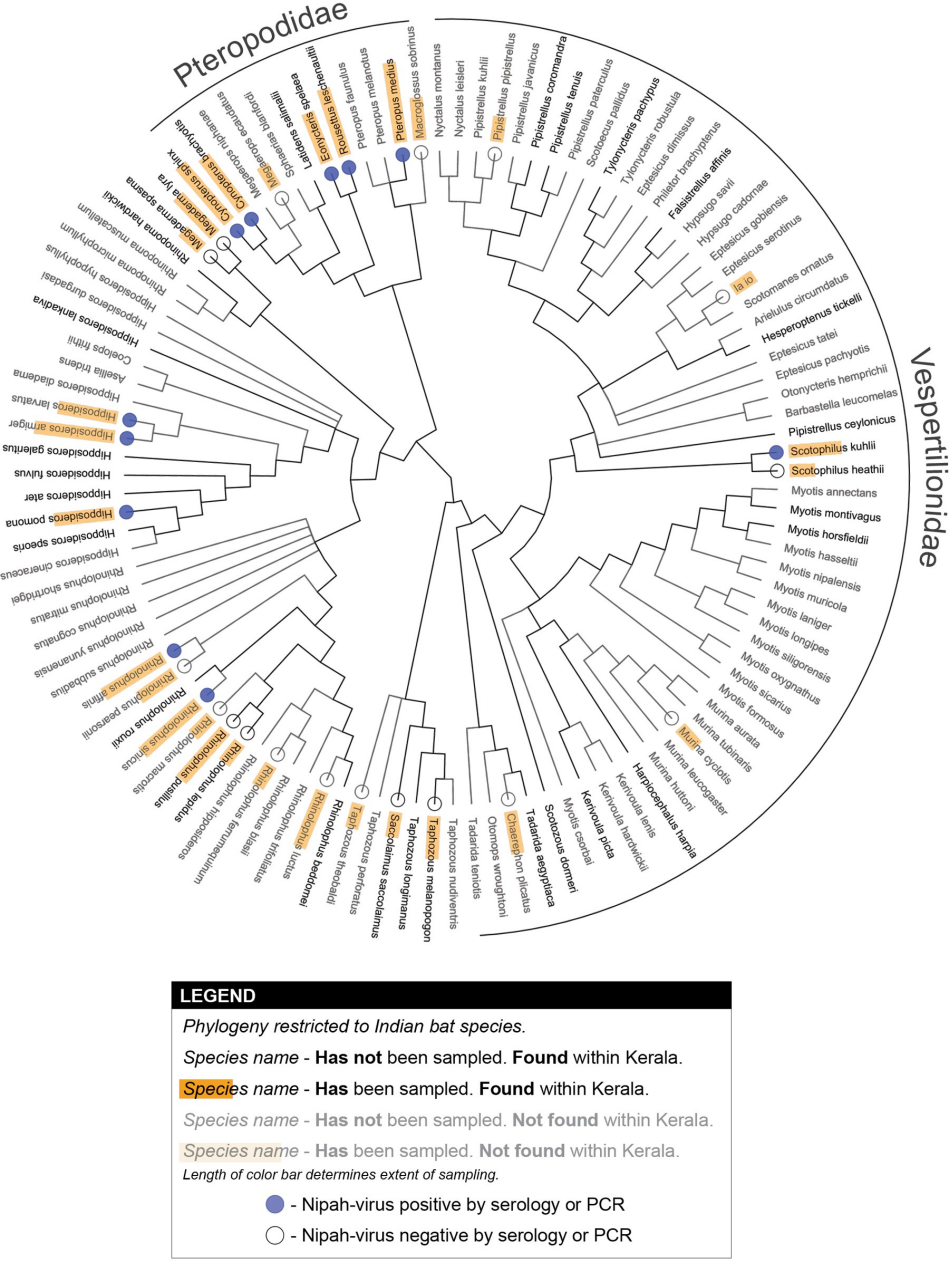
Phylogeny of Indian bats and Nipah virus detections. Serological detections of Nipah virus are scattered across the bat phylogeny, although sampling coverage across the phylogeny is low (see also Table 1). Note that serological assays for Nipah virus likely cross-react with Nipah-like henipaviruses.

As part of a broader study on filoviruses and henipaviruses in wild bats, we systematically searched Web of Science, Centre for Agriculture and Biosciences International (CAB) Abstracts, and PubMed with the following terms: (bat* OR Chiroptera*) AND (filovirus OR henipavirus OR "Hendra virus" OR "Nipah virus" OR "Ebola virus" OR "Marburg virus" OR ebolavirus OR marburgvirus) NOT (human); we also performed a secondary search that included “human”. We followed a systematic exclusion protocol [37] and, because the search was conducted during a study on viral detection or serological detection estimates, we only retained records from observational studies that measured the proportion of wild bats positive for each viral group as assessed by PCR (prevalence) or serology (seroprevalence). We supplemented these data with studies referenced in the systematically identified publications that report viral isolation but not prevalence or seroprevalence. For the generalized boosted regression analysis, we culled the global data by including only studies that reported Nipah virus (by serology or PCR). This search yielded 286 records from 25 papers. For each record, we classified the species, country of sampling, diagnostic method (PCR or serology), sample size, sampling and reporting method (single or multiple cross-sectional events, samples pooled to one estimate), and the proportion of PCR-positive or seropositive bats (Fig 1). We display these data in a phylogenetic context using the bat phylogeny derived from the Open Tree of Life and the *rotl* and *ape* packages (Fig 2) [38, 39].

**Table 1:** Indian bat species that have been sampled for Nipah virus, presence within Kerala, country where sampling occurred, diagnostic methods, sample size, prevalence/seroprevalence and source of data.

Distribution includes Kerala	Species	Country where sampled	Study type^a^	Diagnostic method	Sample size	Proportion of positive samples	Citation
Yes	*Cynopterus brachyotis*	Cambodia	pooled events	serology	1	0	Reynes et al. 2005
		Indonesia	unclear	serology	4	0	Sendow et al. 2006
		Indonesia	unclear	serology	11	0	Sendow et al. 2006
		Malaysia	pooled events	serology	11	0	Kashiwazaki et al. 2004
		Malaysia	pooled events	serology	56	0.04	Johara et al. 2001
	*Cynopterus sphinx*	India	pooled events	serology	30	0	Yadav et al. 2012
		India	pooled events	PCR	30	0	Yadav et al. 2012
		Cambodia	pooled events	serology	68	0	Reynes et al. 2005
		China	pooled events	serology	2	0	Li et al. 2008
		Thailand	pooled events	serology	10	0	Wacharapluesadee et al. 2005
		Vietnam	single event	serology	109	0	Hasebe et al. 2012
		Vietnam	single event	serology	109	0.03	Hasebe et al. 2012
	*Eonycteris spelaea*	Malaysia	pooled events	serology	120	0	Kashiwazaki et al. 2004
		Malaysia	pooled events	serology	38	0.05	Johara et al. 2001
		Thailand	pooled events	serology	54	0	Wacharapluesadee et al. 2005
	*Hipposideros pomona*	Cambodia	pooled events	serology	2	0	Reynes et al. 2005
		China	pooled events	serology	39	0.03	Li et al. 2008
		China	pooled events	serology	20	0	Li et al. 2008
		China	pooled events	serology	1	0	Li et al. 2008
	*Megaderma lyra*	India	pooled events	serology	70	0	Yadav et al. 2012
		India	pooled events	PCR	79	0	Yadav et al. 2012
		China	pooled events	serology	1	0	Li et al. 2008
	*Pteropus medius*^b^	India	single event	serology	29	0.44	Epstein et al. 2008
		India	single event	serology	12	0.67	Epstein et al. 2008
		India	single event	serology	41	0.51	Epstein et al. 2008
		India	single event	serology	29	0.55	Epstein et al. 2008
		India	single event	serology	12	0.83	Epstein et al. 2008
		India	single event	serology	41	0.63	Epstein et al. 2008
		India	pooled events	serology	31	0.03	Yadav et al. 2012
		India	pooled events	PCR	31	0.03	Yadav et al. 2012
		Bangladesh	single event	serology	19	0.11	Hsu et al. 2004
	*Rhinolophus lepidus*	Malaysia	pooled events	serology	1	0	Johara et al. 2001
	*Rhinolophus pusillus*	China	pooled events	serology	1	0	Li et al. 2008
		China	pooled events	serology	7	0	Li et al. 2008
		China	pooled events	serology	7	0	Li et al. 2008
		China	pooled events	serology	11	0	Li et al. 2008
		China	pooled events	serology	9	0	Li et al. 2008
	*Rousettus leschenaultii*	Cambodia	pooled events	serology	15	0	Reynes et al. 2005
		China	pooled events	serology	36	0	Li et al. 2008
		China	pooled events	serology	16	0.31	Li et al. 2008
		Vietnam	single event	serology	74	0.03	Hasebe et al. 2012
		Vietnam	single event	serology	74	0.42	Hasebe et al. 2012
		Thailand	pooled events	serology	4	0	Wacharapluesadee et al. 2005
	*Scotophilus heathi*	Thailand	pooled events	serology	3	0	Wacharapluesadee et al. 2005
	*Scotophilus kuhlii*	Cambodia	pooled events	serology	98	0	Reynes et al. 2005
		China	pooled events	serology	20	0	Li et al. 2008
		Malaysia	pooled events	serology	33	0.03	Johara et al. 2001
	*Taphozous melanopogon*	Malaysia	pooled events	serology	4	0	Johara et al. 2001
		Cambodia	pooled events	serology	69	0	Reynes et al. 2005
	*Taphozous saccolaimus*	Malaysia	pooled events	serology	1	0	Johara et al. 2001
No^c^	*Murina cyclotis*	Cambodia	pooled events	serology	1	0	Reynes et al. 2005
	*Pipistrellus pipistrellus*	China	pooled events	serology	1	0	Li et al. 2008
	*Rhinolophus luctus*	China	pooled events	serology	1	0	Li et al. 2008
		China	pooled events	serology	1	0	Li et al. 2008
		China	pooled events	serology	9	0	Li et al. 2008
		Cambodia	pooled events	serology	1	0	Reynes et al. 2005
	*Taphozous theobaldi*	Cambodia	pooled events	serology	121	0	Reynes et al. 2005
No	*Chaerephon plicatus*	Cambodia	pooled events	serology	153	0	Reynes et al. 2005
		Thailand	pooled events	serology	13	0	Wacharapluesadee et al. 2005
	*Hipposideros larvatus*	Cambodia	pooled events	serology	81	0	Reynes et al. 2005
		Thailand	pooled events	serology	74	0.01	Wacharapluesadee et al. 2005
	*Hipposideros armiger*	Cambodia	pooled events	serology	1	0	Reynes et al. 2005
		China	pooled events	serology	10	0.2	Li et al. 2008
		China	pooled events	serology	11	0	Li et al. 2008
		China	pooled events	serology	5	0	Li et al. 2008
		China	pooled events	serology	12	0	Li et al. 2008
		China	pooled events	serology	4	0	Li et al. 2008
		China	pooled events	serology	1	0	Li et al. 2008
		China	pooled events	serology	20	0	Li et al. 2008
		China	pooled events	serology	20	0	Li et al. 2008
		China	pooled events	serology	1	0	Li et al. 2008
		Thailand	pooled events	serology	6	0	Wacharapluesadee et al. 2005
	*Ia io*	China	pooled events	serology	7	0	Li et al. 2008
	*Macroglossus sobrinus*	Cambodia	pooled events	serology	1	0	Reynes et al. 2005
		Malaysia	pooled events	serology	4	0	Johara et al. 2001
	*Megaerops ecaudatus*	Malaysia	pooled events	serology	1	0	Johara et al. 2001
	*Rhinolophus affinis*	China	pooled events	serology	26	0.04	Li et al. 2008
		China	pooled events	serology	1	0	Li et al. 2008
		China	pooled events	serology	48	0	Li et al. 2008
		China	pooled events	serology	17	0	Li et al. 2008
		China	pooled events	serology	2	0	Li et al. 2008
		Malaysia	pooled events	serology	6	0	Johara et al. 2001
	*Rhinolophus ferrumequinum*	China	pooled events	serology	3	0	Li et al. 2008
	*Rhinolophus macrotis*	China	pooled events	serology	3	0	Li et al. 2008
	*Rhinolophus pearsonii*	China	pooled events	serology	32	0	Li et al. 2008
		China	pooled events	serology	3	0	Li et al. 2008
	*Rhinolophus sinicus*	China	pooled events	serology	15	0.07	Li et al. 2008
		China	pooled events	serology	17	0	Li et al. 2008
		China	pooled events	serology	5	0	Li et al. 2008
		China	pooled events	serology	1	0	Li et al. 2008
		China	pooled events	serology	9	0	Li et al. 2008
		China	pooled events	serology	3	0	Li et al. 2008
		China	pooled events	serology	1	0	Li et al. 2008

^a^pooled events report results from multiple time points as a single estimate

^b^formerly known as *Pteropus giganteus*

^c^The IUCN distribution maps erroneously include *R*. *luctus*, *M*. *cyclotis*,*T*. *theobaldi*, and *P*. *pipistrellus* in Kerala; however, these species are not found in Kerala [58, PO Nameer personal communication]

### Machine learning analyses

To make predictions of bat species that may carry Nipah virus in India and the surrounding region, we trained a generalized boosted regression model on data that characterized 48 traits of 523 extant bat species with geographic ranges in Asia, Australia, and Oceana. By learning the intrinsic features of species that have previously been found to have evidence of Nipah virus- infection (in this study, either through serology or PCR), the objective is to identify additional bat species whose trait profiles suggest a high probability of being Nipah virus-positive. In addition, by examining those traits that are most predictive of Nipah virus-positive species, we may also glean ecological insights about why some bats are found to be Nipah virus-positive compared to others in this region. While examination of these suites of shared traits can be insightful, it is important to note that these methods are designed for pattern recognition rather than to identify mechanisms; however, in some cases, mechanisms may be suggested [42]).

We acquired range maps from the International Union for Conservation of Nature (IUCN) [43]. We obtained data on foraging method and diet composition from EltonTraits [44]. We derived data on biological and ecological attributes from PanTHERIA [45]. We took data on torpor and migration behaviors from Luis et al. [46], and data on production (a measure of fitness output) from Hamilton et al. [47]. All variables, their definitions, coverage, and data source citations are reported in S1 Table. Models were trained on 80% of this full data set and comprised of 50,000 trees specifying a Bernoulli error distribution and built with 10-fold cross-validation to prevent overfitting. In addition, we weighted each species by its sample size (“sum.sample.size”) to account for the fact that some species are more frequently sampled for henipaviruses compared to others. We also applied target shuffling methods to calculate the corrected area under the curve (AUC) [48].

We conducted a second generalized boosted regression analysis to diagnose whether greater data availability for better-studied species leads to trait profiles that describe well studied bat species rather than species where evidence of Nipah virus infection has been reported. In this model, we used the number of citations in Web of Science for each species’ scientific name as a proxy for study effort at the time this study was conducted. As before, models were trained on 80% of the full data set and were comprised of 30,000 trees specifying a Poisson error distribution and built with 10-fold cross-validation to prevent overfitting. Hyperparameter values and outputs for generalized boosted regression models can be found in S2 Table.

## Results

### Previous surveys

One hundred twelve species of bats have been detected in India, of which 39 have been detected within the state of Kerala [43, 49, 50]. Thirty-one bat species that occur in India (and 18 that occur in Kerala) have been sampled for Nipah virus and 11 of these species have been identified as having antibodies that react to Nipah virus serological tests. However, almost all sampling of these species occurred outside of India. The 11 positive species include seven species that reside in Kerala, including five Pteropodidae (*Cynopterus brachyotis*, *C*. *sphinx*, *Eonycteris spelaea*, *Rousettus leschenaultii*, and *P*. *medius* [formerly *P*. *giganteus*]) and two non-Pteropodidae (*Scotophilus kuhlii* and *Hipposideros pomona*; Table 1 [30, 40, 41, 51–56]). Although all of these species had serological evidence of Nipah virus (or cross-reacting Nipah-like viruses), *P*. *medius* was the only species with virological evidence of Nipah virus (1 out of 31 individuals tested with PCR [3%]) [40, 41]. Seroprevalence in sampled species ranged from 0–83% and prevalence from 0–3% (Table 1). *P*. *medius* [41] and *R*. *leschenaultia* [56, 57] were the only species with seroprevalence >30%. However, most studies reported seroprevalence as pooled detection over time (i.e. samples from multiple time points were included in a single seroprevalence estimate). Only three species (*P*. *medius*, *Cynopterus sphinx*, *and Megaderma lyra*) were sampled within India, and one of these species (*P*. *medius*) had evidence of viral shedding within India [40, 41] (Table 1 and Fig 2). Recent media reports suggest that additional cross-sectional surveys of bats have been conducted in response to the outbreak in Kerala and that *P*. *medius* tested positive by PCR [14].

In Fig 2, we map detections of Nipah virus by serology or PCR onto the phylogeny of bat species found in India. Our qualitative assessment of Nipah virus detections among these species, within a phylogenetic context, suggested clustering of Nipah virus positivity within Pteropodidae, consistent with the ongoing focus of research efforts on this family. However, Nipah virus reactivity was also detected in other bat families (Fig 2). Moreover, some clades that contain henipavirus-seropositive bats also contain species that occur in Kerala but have not been sampled (Fig 2). For example, a number of unsampled *Hipposideros* and *Rhinolophus* that occur in Kerala are members of clades that include Nipah-virus seropositive bats (Fig 2).

### Likely reservoirs

**Fig 3:**
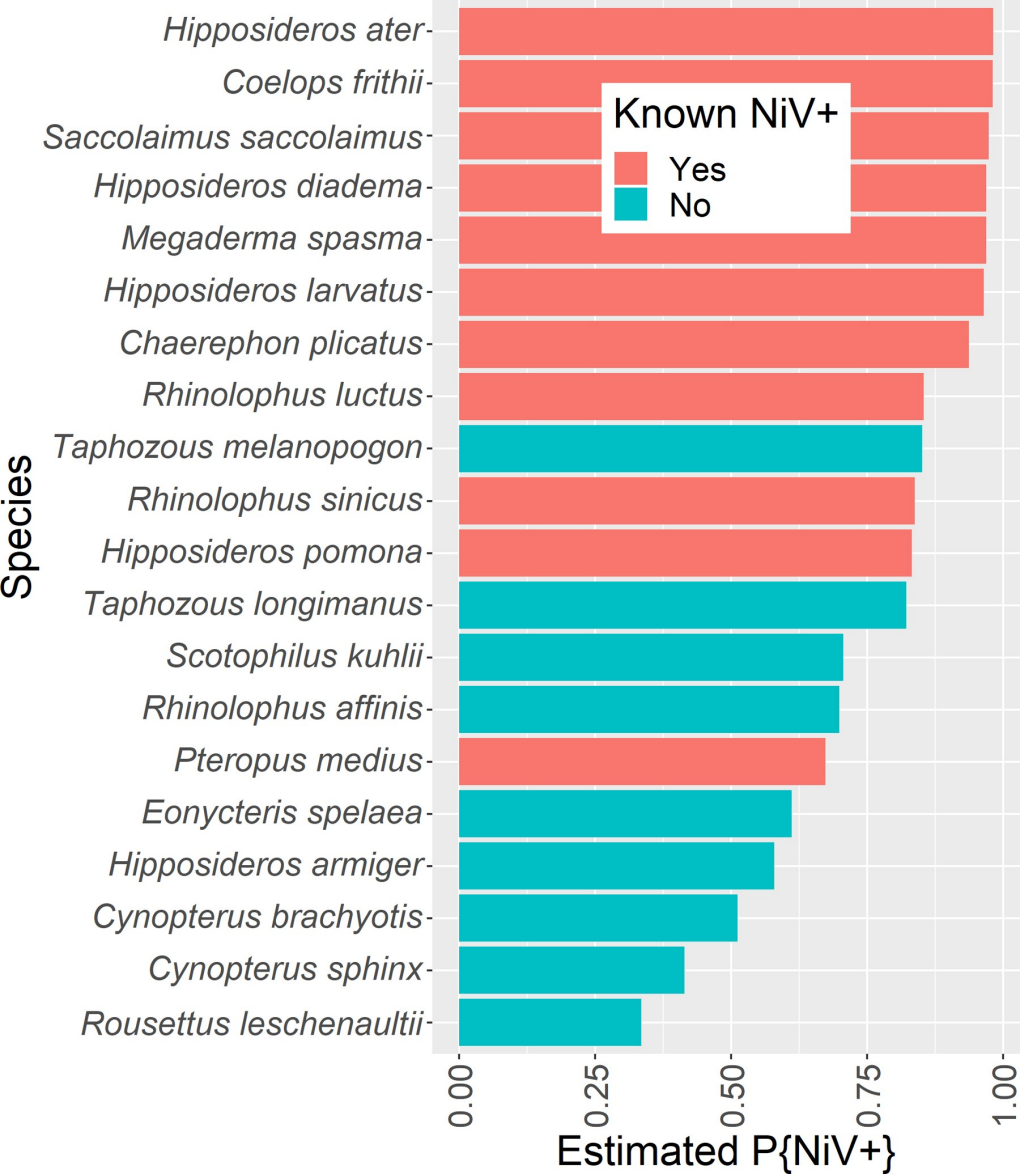
Predicted probability of top 20 Indian bat species being Nipah virus positive. Nipah virus has been detected in *Pteropus medius*, but other bat species have either known exposure (serological reactivity to Nipah virus) or predicted exposure based on our analysis of Nipah virus surveys. Red indicates having evidence of Nipah virus exposure or infection (by serology or PCR) and blue indicates no previous evidence of Nipah virus exposure.

**Fig 4:**
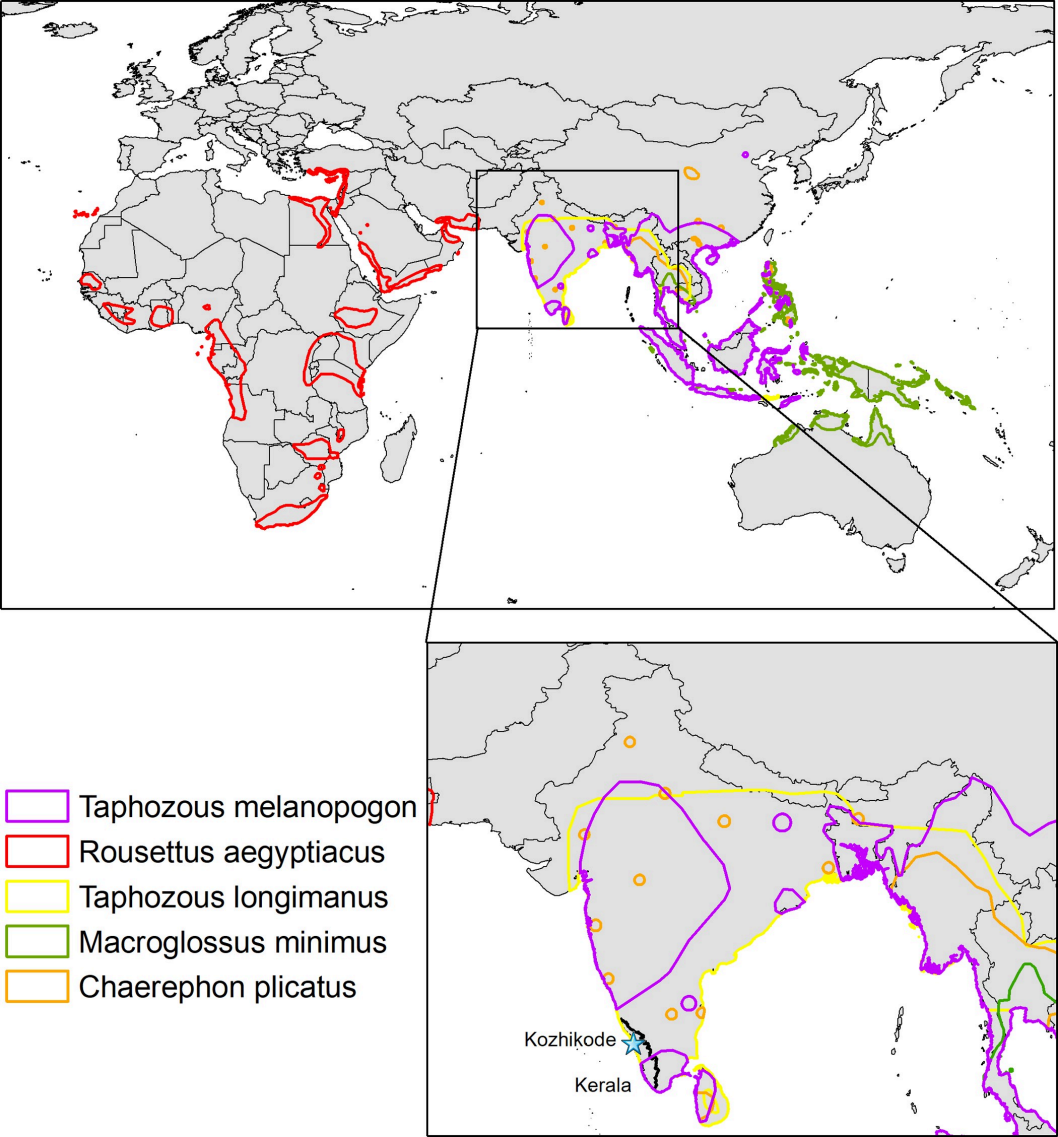
Range of predicted bat species. Geographic ranges of bat species that are in the 90^th^ percentile of similarity (based on generalized boosted regression) with other bat species that are positive for Nipah virus from Asia, Australia, and Oceana (based on PCR or serology). The terrestrial mammal range shapefile was downloaded from the IUCN website (http://www.iucnredlist.org/technical-documents/spatial-data) and the figure was created with ArcGIS.

The generalized boosted regression model that we applied to species-level trait data identified Nipah virus-positive bat species with ~83% accuracy (Fig 3; corrected AUC = 0.83; complete model outputs and hyperparameters are reported in S2 and S3 Tables). In addition to Nipah virus-positive bat species, we identified six species with geographic ranges overlapping Asia, Australia, and Oceana that are not currently identified as Nipah reservoirs but, on the basis of trait similarity with known Nipah virus-seropositive or virological-positive bat species, have high likelihood of exposure to Nipah virus: *Rousettus aegyptiacus*, *Taphozous longimanus*, *Taphozous melanopogon*, *Rhinolophus luctus*, *Chaerophon plicatus*, and *Macroglossus minimus*. The geographic ranges of four of these species overlap with India: *C*. *plicatus*, *R*. *luctus*, *T*. *longimanus*, and *T*. *melanopogon*. The latter two species overlap with Kerala, with probabilities of Nipah virus-positivity ~80%. (S3 Table and Fig 4; note that IUCN distribution maps erroneously include *R*. *luctus*, *Murina cyclotis*, *Taphozous theobaldi*, and *Pipistrellus pipistrellus* in Kerala; however, these species are not found in Kerala [58, PO Nameer personal communication, 59]). Study effort was not predictable on the basis of traits, suggesting that the trait profile of bat species that are Nipah virus-positive are not confounded by traits simply associated with well-studied species. Model hyperparameters, performance metrics, and relative importance scores for all traits are available in S2 Table. Citation counts for each species are available in the data repository.

## Discussion

Our trait-based analyses identified four additional Indian bat species to target for surveillance for Nipah virus; two of these species occur within Kerala. Our predictions inform a research pipeline that should include serosurveys of these potential bat reservoirs and the 11 Indian bat species previously identified to have evidence of Nipah virus infection. Species that are seropositive on these initial surveys should then undergo longitudinal spatiotemporal surveillance to detect shedding. Our predictions must be combined with local knowledge on bat ecology—including distribution, abundance, and proximity to humans—to design sampling plans that can effectively identify hosts that pose a risk to humans [60]. Moreover, sampling of bats should be combined with epidemiological, anthropological, ecological, immunological, and virological work to uncover the relations that drive transmission of virus from animals to humans.

Nipah virus has a wide host breadth in both reservoir bat species and recipient animal species. Therefore, identifying the reservoir in a new location can be challenging. We used a systematic literature search to collate data from previous studies of Nipah virus in bats. We then prioritized surveillance of bats in Kerala, and more generally in India, on the basis of these data. We applied a trait-based generalized boosted regression that identified species with traits similar to those associated with serological or virological evidence of Nipah virus. Nipah virus was detected by PCR in only one species occurring in India, *P*. *medius*, which also is the known reservoir in Bangladesh. However, Nipah virus was detected by serology in many species. Eleven out of 112 bat species that occur in India, and seven of the 39 species that occur in Kerala, had serological evidence of Nipah virus exposure (most were sampled outside of India).

Our work provides a list of species to guide early surveillance and should not be taken as a definitive list of reservoirs. A series of further studies are required to triangulate on the reservoir hosts that pose a risk to humans. A major reason these studies do not identify definitive reservoirs is because almost all previous Nipah virus studies relied on serology, but serological assays often lack specificity; detection of Nipah virus may represent cross-reactions to closely related viruses [61]. For example, multiple studies have shown cross reactivity among Hendra, Cedar, and Nipah viruses using glycoprotein assays [62–64]. It is likely that many of the positive tests reported here represent exposure to uncharacterized henipaviruses with antigenic similarity to Nipah virus. These viruses may or may not be zoonotic. PCR is specific and sensitive, and positive results demonstrate presence of Nipah virus RNA; however, the prevalence of Nipah virus is usually so low that large sample sizes are needed to yield positive detections [27, 65] outside of pulses of shedding [29, 36]). Therefore, PCR may not be informative in the early stages of identifying reservoirs. Serology remains an important tool for these initial surveys as long as the assays are interpreted correctly, and positive detections are followed by virological studies to detect shedding. These field surveys need to be followed by virological studies to characterize viruses and their zoonotic risk and then epidemiological studies to understand risk to public health [61].

In addition to suggesting potential reservoir species, the associative traits that predict reservoir capacity inform the ecology of potential bat reservoirs, which may guide epidemiological studies of Nipah virus infection. However, the utility of these traits as predictors of reservoir capacity should be interpreted as associative rather than causal. Some of the traits in the generalized boosted regression (see Supporting Information S2 Table) capture potential phylogenetic structure of Nipah virus hosts. For example, the relative importance of adult body length and forearm length could reflect the strong association of Nipah virus with medium to large Pteropodidae bats, although 'Pteropodidae' was not itself an important predictor (S2 Table). Beyond including bat families as taxonomic predictor variables, our analysis largely subsumes additional phylogenetic structure underlying patterns of Nipah virus seropositivity in bat species. It is likely that patterns of evolutionary relatedness among host species may underlie similarities in factors that determine host receptivity. Such factors may include functional receptors that enable viral entry into host cells and host factors required for viral replication [66, 67]. Patterns of co-divergence of hosts and viruses [68] are also reflected in host and viral phylogeny. The association of these traits with reservoir capacity should be elucidated by future phylogenetic comparative analyses of host traits, which will rely on expanded availability of relevant data (e.g., characterization of species level differences in functional receptors).

Other traits with high relative influence included aridity (mean precipitation [mm]/mean potential evapotranspiration [mm]), the maximum latitudinal extent of each species geographic range, the richness of mammal species found within a species’ geographic range, and the trophic level of each species (see S2 Table, and partial dependence plots, S1 Fig). In general, our analysis suggests Nipah virus-positive bats in this region tend to be herbivorous or omnivorous species whose geographic ranges overlap with tropical desert (arid) habitats, maximally extending to the northern limit of the tropical belt and overlapping with a high diversity of other mammal species (S1 Fig). Given that bats from arid habitats may forage more widely when water or food resources become limited in dry years, it is also possible that Nipah virus transmission may occur with increasing contact between multiple bat species mixing at higher densities around limited resources [24].

A current constraint on progress towards understanding the epidemiology of Nipah virus in India is the dearth of virologic and taxonomic studies on bats in India. The majority of studies used for these analyses were conducted outside of India and no studies, to our knowledge, investigated Nipah virus in Kerala prior to this outbreak. India encompasses many different bioregions. The outbreak in Kerala shows that the ecological niche for Nipah virus is very wide and could include the entire distribution of *P*. *medius*, as well as the distributions of other potential reservoirs proposed here. Studies in wildlife and humans must cover this broad geography to assess future risk in India. Moreover, the last comprehensive and systematic taxonomic study on the bats in India was conducted more than a century ago. There are several cryptic species or species with unresolved taxonomic status in India, and it is possible that species with Nipah virus detections outside of India may have been misidentified. Therefore, our conclusions may change after detailed and systematic taxonomic studies are done on Indian bats.

Once serological evidence of Nipah virus is detected in potential reservoir hosts, longitudinal spatial and temporal surveillance of these hosts will be necessary. Detection of virus at a single point in time and space conveys limited information and could represent a spillover event from another species. To confirm reservoirs status of a species, virus must be consistently found within that species [69]. Moreover, maintenance of henipaviruses can be extremely dynamic. Seasonal, annual, interannual, or stochastic pulses of shedding can be driven by extinction and recolonization of virus among bat populations or episodic shedding in response to stress (see discussions in [26]). Therefore, discriminating viral maintenance versus spillover, and characterizing shedding dynamics, requires intensive sampling over time and space.

Identifying reservoir hosts and then characterizing the diversity of their viruses and their virus shedding patterns are critical steps in understanding spillover. However, the transmission of Nipah virus from bats to humans requires alignment of a number of other ecological and epidemiological factors [67], including bat and human behaviors that expose humans to an infectious dose of Nipah virus. In Bangladesh and Australia, bat and human behaviors facilitate exposure to Nipah and Hendra virus, respectively, when bats exploit human food. In Bangladesh, bats contaminate human-harvested date palm sap [7]. In Australia, bats exploit food from trees in peri-urban areas when native winter food sources are cleared [26, 70]. When pulses of virus shedding in bats coincide with bat and human or horse contact through food, spillover is more likely to occur [71]. Understanding these important interfaces requires a variety of epidemiological studies including niche and spatial risk modeling [72], as well as animal and human behavioral studies [7, 11].

In addition to sampling bat reservoir hosts, sampling plans should consider that henipaviruses could be maintained in domestic recipient hosts. These hosts, with closer and more frequent contact with humans, can become bridge hosts for human infections [36]. For example, Nipah virus was repeatedly introduced into intensive commercial pig populations in Malaysia. These repeated introductions of Nipah virus into pig farms allowed accumulation of herd immunity and the conditions for long term persistence and regional spread that facilitated transmission to humans [10]. To narrow potential spillover pathways to humans in India, studies should consider susceptible domestic animal species with husbandry that facilitates virus persistence (e.g., intensive commercial farming systems with high turnover of animals).

Projecting the risk of Nipah virus outbreaks in humans requires identification of the reservoir hosts and the dynamics of Nipah virus within those hosts. Our predictions inform initial sampling that can be followed by a sequence of studies that investigate the bat species highlighted here. The machine learning approaches presented here can be the first step in a research pipeline to eventually understand the mechanisms underpinning epidemiologically important cross-species contacts.

## Data availability

Data is available at https://caryinstitute.figshare.com/s/8f79fff6795132cabc1a

## Supporting information

S1 Table Variables in generalized boosted regression analyses. All variables included in the generalized boosted regression analyses, together with the coverage of each variable across species, and how each variable is defined according to source references. Variable names are consistent with those reported in original citations.
(PDF)

S2 Table The hyperparameter values and resulting trait profiles of two generalized boosted regression analyses on bats species from Asia, Australia, and Oceana. Results of the first analysis (columns B and C) treat Nipah virus positivity as a binary response variable. Results of the second analysis (columns D and E) treat study effort as a Poisson distributed response variable (the number of citations returned from a search in Web of Science on the Latin binomials of each species).
(PDF)

S3 Table Species predictions. A list of bat species in descending order NiV positivity as predicted by a generalized boosted regression model on species-level traits (pNiV+), and a binary column (NiV+) indicating seropositivity from all previously published work.
(PDF)

S1 Fig Partial dependence plots of the top six most important features for NiV classification accuracy. The frequency histogram of trait value distribution across all bat species (blue bars) is overlaid with a black line indicating model sensitivity to trait values for classification accuracy.
(PDF)

S1 Checklist PRISMA checklist. (PDF)

S2 Checklist Included studies. Table of included papers.
(PDF)

S1 Diagram PRISMA flow diagram. (PDF)
