# Correction: Prioritizing surveillance of Nipah virus in India

**DOI:** 10.1371/journal.pntd.0011126

**Published:** 2023-02-10

**Authors:** Raina K. Plowright, Daniel J. Becker, Daniel E. Crowley, Alex D. Washburne, Tao Huang, P. O. Nameer, Emily S. Gurley, Barbara A. Han

The order of species names in Fig 3 is incorrect. The authors have provided a corrected version here.

**Fig 3:**
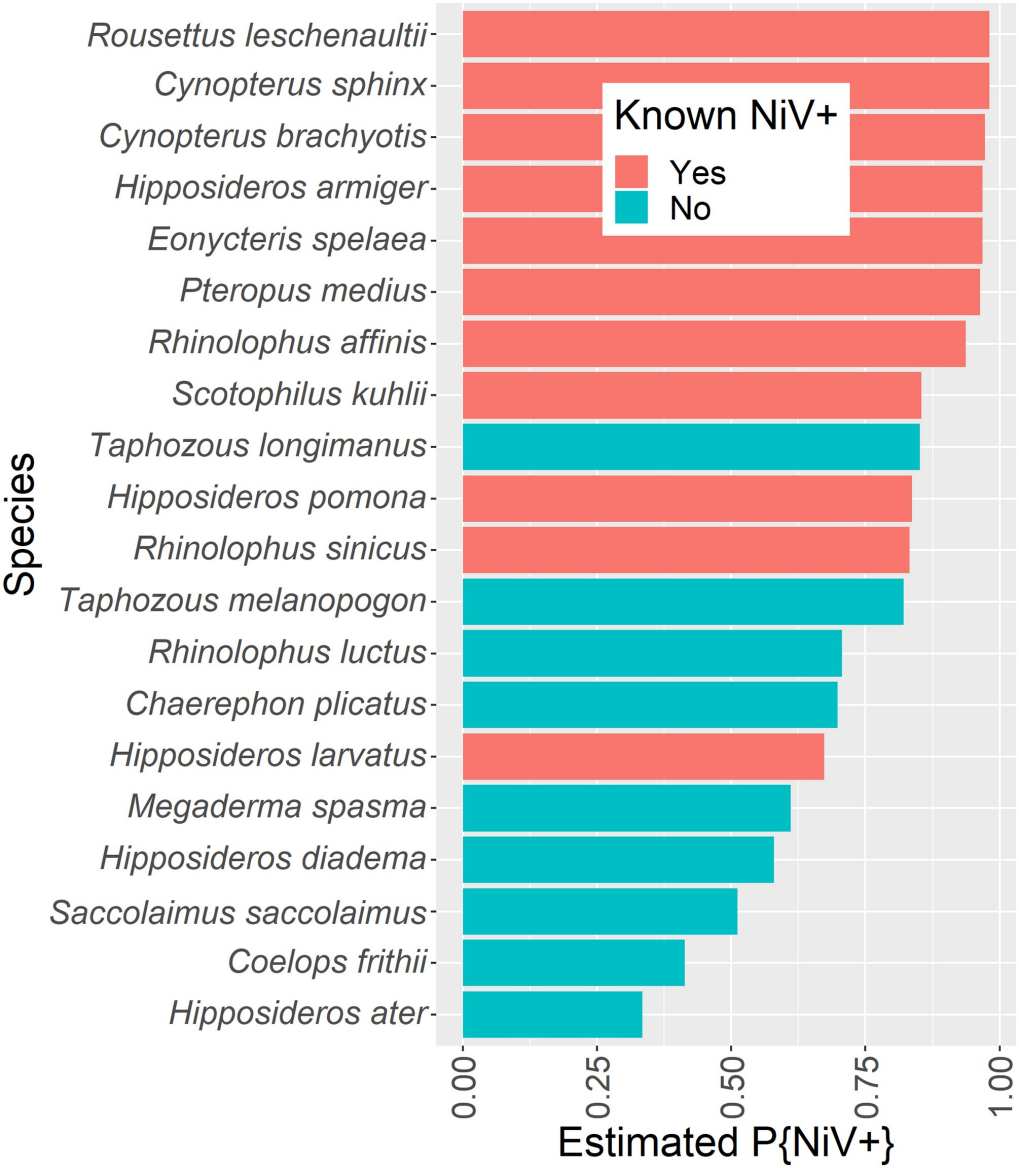
Predicted probability of top 20 Indian bat species being Nipah virus positive. Nipah virus has been detected in *Pteropus medius*, but other bat species have either known exposure (serological reactivity to Nipah virus) or predicted exposure based on our analysis of Nipah virus surveys. Red indicates having evidence of Nipah virus exposure or infection (by serology or PCR) and blue indicates no previous evidence of Nipah virus exposure.
