# Supplementary material for: Prioritizing surveillance of Nipah virus in India
Source: PLoS Negl Trop Dis. 2019 Jun 27;13(6):e0007393. doi: 10.1371/journal.pntd.0007393 (PMC6597033; doi:10.1371/journal.pntd.0007393)
Supplement: S1 Table — All variables included in the generalized boosted regression analyses, together with the coverage of each variable across species, and how each variable is defined according to source references. Variable names are consistent with those reported in original citations. (PDF) [file pntd.0007393.s001.pdf]

S1 Table. Variables in generalized boosted regression analyses

Predictor	Coverage	Definition	Source
AnnualBP	0.017	The average number of birth pulses per year for the species	Luis et al. 2013
X1.1_ActivityCycle	0.057	Activity cycle of each species measured for non-captive populations; adult or age unspecified individuals, male, female, or sex unspecified individuals; primary, secondary, or extrapolated sources; all measures of central tendency; in all localities. Species were defined as (1) nocturnal only, (2) nocturnal/crepuscular, cathemeral, crepuscular or diurnal/crepuscular and (3) diurnal only.	Jones et al. 2008 PanTHERIA
X5.1_AdultBodyMass_g	0.623	Mass of adult (or age unspecified) live or freshly-killed specimens (excluding pregnant females) using captive, wild, provisioned, or unspecified populations; male, female, or sex unspecified individuals; primary, secondary, or extrapolated sources; all measures of central tendency; in all localities.	Jones et al. 2008 PanTHERIA
X8.1_AdultForearmLen_mm	0.805	Total length from elbow to wrist of adult (or age unspecified) live, freshly-killed, or museum specimens using captive, wild, provisioned, or unspecified populations; male, female, or sex unspecified individuals; primary, secondary, or extrapolated sources; all measures of central tendency; in all localities.	Jones et al. 2008 PanTHERIA
X13.1_AdultHeadBodyLen_mm	0.168	Total length from tip of nose to anus or base of tail of adult (or age unspecified) live, freshly-killed, or museum specimens using captive, wild, provisioned, or unspecified populations; male, female, or sex unspecified individuals; primary, secondary, or extrapolated sources; all measures of central tendency; in all localities.	Jones et al. 2008 PanTHERIA
X2.1_AgeatEyeOpening_d	0.036	Age at which both eyes are fully open after birth using captive, wild, provisioned, or unspecified populations; male, female, or sex unspecified individuals; primary, secondary, or extrapolated sources; all measures of central tendency; in all localities	Jones et al. 2008 PanTHERIA
X3.1_AgeatFirstBirth_d	0.073	Age at which females give birth to their first litter (eutherians), or their young attach to teats (metatherians) or hatch out (monotremes), using non-captive, wild, provisioned, or unspecified populations; primary, secondary, or extrapolated sources; all measures of central tendency; in all localities.	Jones et al. 2008 PanTHERIA
X18.1_BasalMetRate_mLO2hr	0.044	Basal metabolic rate of adult (or age unspecified) individual(s) using captive, wild, provisioned, or unspecified populations; male, female, or sex unspecified individuals; primary, secondary, or extrapolated sources; all measures of central tendency; in all localities. Metabolic rate was measured when individual(s) were experiencing neither heat nor cold stress (i.e. are in their thermoneutral zone); are resting and calm; and are post–absorptive (are not digesting or absorbing a meal) and data were only accepted where there was also a measure of body mass for the same individual(s).	Jones et al. 2008 PanTHERIA
X5.2_BasalMetRateMass_g	0.044	Mass of individual(s) from which the basal metabolic rate was taken.	Jones et al. 2008 PanTHERIA
X6.1_DietBreadth	0.377	Number of dietary categories eaten by each species measured using any qualitative or quantitative dietary measure, over any period of time, using any assessment method, for non-captive or non-provisioned populations; adult or age unspecified individuals, male, female, or sex unspecified individuals; primary, secondary, or extrapolated sources; all measures of central tendency; in all localities. Categories were defined as vertebrate, invertebrate, fruit, flowers/nectar/pollen, leaves/branches/bark, seeds, grass and roots/tubers.	Jones et al. 2008 PanTHERIA
X9.1_GestationLen_d	0.130	Length of time of non-inactive fetal growth, using captive, wild, provisioned, or unspecified populations; male, female, or sex unspecified individuals; primary, secondary, or extrapolated sources; all measures of central tendency; in all localities. Gestation was measured between specified start and end points as follows: Start points – conception, fertilization, first observed copulation, fertilization, implantation, laying, palpably pregnant, removal of pouch young, capture (except marsupials) or unspecified. End points – birth, hatching or unspecified.	Jones et al. 2008 PanTHERIA
X15.1_LitterSize	0.352	Number of offspring born per litter per female, either counted before birth, at birth or after birth, using captive, wild, provisioned, or unspecified populations; male, female, or sex unspecified individuals; primary, secondary, or extrapolated sources; all measures of central tendency; in all localities.	Jones et al. 2008 PanTHERIA
X16.1_LittersPerYear	0.187	Number of litters per female per year using non-captive, wild, provisioned, or unspecified populations; male, female, or sex unspecified individuals; primary, secondary, or extrapolated sources; all measures of central tendency; in all localities.	Jones et al. 2008 PanTHERIA
X17.1_MaxLongevity_m	0.061	Maximum adult age measured either through direct observation, capture-recapture estimates, projected from physical wear or unspecified, using captive, wild, provisioned, or unspecified populations; male, female, or sex unspecified individuals; primary, secondary, or extrapolated sources; in all localities.	Jones et al. 2008 PanTHERIA
X5.3_NeonateBodyMass_g	0.138	Mass of live or freshly-killed specimens of infants at either a near term embryonic stage, birth, immediately after birth or up to an age of seven days after birth, using captive, wild, provisioned, or unspecified populations; male, female, or sex unspecified individuals; primary, secondary, or extrapolated sources; all measures of central tendency; in all localities.	Jones et al. 2008 PanTHERIA
X13.2_NeonateHeadBodyLen_mm	0.036	Total length from tip of nose to anus or base of tail of live, freshly-killed, or museum specimens of infants at either a near term embryonic stage, birth, immediately after birth or up to an age of seven days after birth, using captive, wild, provisioned, or unspecified populations; male, female, or sex unspecified individuals; primary, secondary, or extrapolated sources; all measures of central tendency; in all localities.	Jones et al. 2008 PanTHERIA
X10.1_PopulationGrpSize	0.111	Number of individuals, adults or definition unspecified in a group that spends the majority of their time in a 24 hour cycle together, measured over any duration of time, using non-captive populations; male, female, or sex unspecified individuals; primary, secondary, or extrapolated sources; all measures of central tendency; in all localities.	Jones et al. 2008 PanTHERIA
X23.1_SexualMaturityAge_d	0.109	Age when individuals are first physically capable of reproducing, defined as either physically sexually mature, age at first mating or unspecified (males and females), age at first estrus or age at first pregnancy (females only), age at spermatogenesis or age at testes descent (males only), using captive, wild, provisioned, or unspecified populations; male, female, or sex unspecified individuals, primary, secondary, or extrapolated sources; all measures of central tendency; in all localities.	Jones et al. 2008 PanTHERIA
X6.2_TrophicLevel	0.377	Trophic level of each species measured using any qualitative or quantitative dietary measure, over any period of time, using any assessment method, for non-captive or non-provisioned populations; adult or age unspecified individuals, male, female, or sex unspecified individuals; primary, secondary, or extrapolated sources; all measures of central tendency; in all localities. Species were defined as (1) herbivore (not vertebrate and/or invertebrate), (2) omnivore (vertebrate and/or invertebrate plus any of the other categories) and (3) carnivore (vertebrate and/or invertebrate only)	Jones et al. 2008 PanTHERIA
X25.1_WeaningAge_d	0.143	Age when primary nutritional dependency on the mother ends and independent foraging begins to make a major contribution to the offspring's energy requirements, measured as either weaning/lactation length, nutritionally independent, first solid food, last observed nursing, age at first flight (bats only), age at pouch exit or length of teat Attachment (marsupials only) or unspecified definition, using captive, wild, provisioned, or unspecified populations; male, female, or sex unspecified individuals; primary, secondary, or extrapolated sources; all measures of central tendency; in all localities.	Jones et al. 2008 PanTHERIA
X5.4_WeaningBodyMass_g	0.059	Mass of live or freshly-killed specimens of weanlings, using captive, wild, provisioned, or unspecified populations; male, female, or sex unspecified individuals; primary, secondary, or extrapolated sources; all measures of central tendency; in all localities.	Jones et al. 2008 PanTHERIA
X26.1_GR_Area_km2	0.967	Geographic range area, calculated using the total extent of a species range with a global equal-area projection (Mollweide)	Jones et al. 2008 PanTHERIA
X26.2_GR_MaxLat_dd	0.967	The maximum latitudinal extent of each species geographic range calculated using a global geographic projection (decimal degrees)	Jones et al. 2008 PanTHERIA
X26.3_GR_MinLat_dd	0.967	The minimum latitudinal extent of each species range calculated using a global geographic projection (decimal degrees)	Jones et al. 2008 PanTHERIA
X26.4_GR_MidRangeLat_dd	0.967	The median latitudinal extent of each species range calculated using a global geographic projection (decimal degrees)	Jones et al. 2008 PanTHERIA
X26.5_GR_MaxLong_dd	0.967	maximum longitudinal extent of each species range calculated using a global geographic projection (decimal degrees)	Jones et al. 2008 PanTHERIA
X26.6_GR_MinLong_dd	0.967	minimum longitudinal extent of each species range calculated using a global geographic projection (decimal degrees)	Jones et al. 2008 PanTHERIA
X26.7_GR_MidRangeLong_dd	0.967	median longitudinal extent of each species range calculated using a global geographic projection (decimal degrees)	Jones et al. 2008 PanTHERIA
X27.1_HuPopDen_Min_n.km2	0.967	Minimum human population density (persons per km2) using the Gridded Population of the World (GPW) (CIESIN and CIAT 2005) for 1995	Jones et al. 2008 PanTHERIA
X27.2_HuPopDen_Mean_n.km2	0.967	Mean human population density (persons per km2) using the Gridded Population of the World (GPW) (CIESIN and CIAT 2005) for 1995	Jones et al. 2008 PanTHERIA
X27.3_HuPopDen_5p_n.km2	0.967	5th percentile human population density (persons per km2) using the Gridded Population of the World (GPW) (CIESIN and CIAT 2005) for 1995	Jones et al. 2008 PanTHERIA
X27.4_HuPopDen_Change	0.964	Mean rate of increase in human population density using the Gridded Population of the World (GPW) (CIESIN and CIAT 2005) for 1990 and 1995 as: (1995–1990)/1990	Jones et al. 2008 PanTHERIA
X28.1_Precip_Mean_mm	0.927	mean monthly precipitation (mm) calculated using data from ftp://ftp.ngdc.noaa.gov/Solid_Earth/Ecosystems/GEDII_a/datasets/a03/lc.htm	Jones et al. 2008 PanTHERIA
X28.2_Temp_Mean_01degC	0.927	mean monthly temperature (0.1°C) calculated using data from ftp://ftp.ngdc.noaa.gov/Solid_Earth/Ecosystems/GEDII_a/datasets/a03/lc.htm	Jones et al. 2008 PanTHERIA
X30.1_AET_Mean_mm	0.780	mean monthly AET (Actual Evapotranspiration Rate) from 1920 to 1980 (mm) calculated using the Global Resource Information Database of UNEP and is available from http://www.grid.unep.ch/data/grid/gnv183.php	Jones et al. 2008 PanTHERIA
X30.2_PET_Mean_mm	0.780	mean monthly PET (Potential Evapotranspiration Rate) from 1920 to 1980 (mm) calculated using the Global Resource Information Database of UNEP and is available from http://www.grid.unep.ch/data/grid/gnv183.php	Jones et al. 2008 PanTHERIA
Log10.mass_specific_production.	0.052	The log-base 10 of production, where production is the mean mass of offspring produced per year, normalized by adult body size	Hamilton et al. 2010
Diet.Inv	1.000	Percent of diet comprised of invertebrates	Wilman et al. 2014 EltonTraits
Diet.Fruit	1.000	Percent of diet comprised of fruit	Wilman et al. 2014 EltonTraits
BodyMass.Value	1.000	Mean of body mass for both sexes (in g)	Wilman et al. 2014 EltonTraits
torp	0.061	Categorization of torpor use, where 1 = no evidence of torpor use; 2 = some torpor use with minimum body temperature> 11◦C; 3 = true hibernation with min Tb< 11◦C	Luis et al. 2013
mig	0.061	Categorization of migration level, where 1 = only local movement; 2 = regional migration, 3 = long distance migration	Luis et al. 2013
aridity	0.780	Mean Precipitation (mm)/Mean PET (mm)	constructed
BodySizeRatio	0.138	Adult Body Mass (g)/Neonate Body Mass (g)	constructed
postnatGR	0.054	Weaning body mass/neonatal body mass	constructed
RelAgeSexMat	0.042	Age at sexual maturity/maximum longevity	constructed
sppdens	0.950	The richness of mammal species found within a species' geographic range	constructed using IUCN Terrestrial Mammals http://www.iucnredlist.org/technical-documents/spatial-data
spp.dens.per.km	0.920	The richness of mammal species found within a species' geographic range divided by the geographic range area in units of n per kilometer2	constructed using IUCN Terrestrial Mammals http://www.iucnredlist.org/technical-documents/spatial-data

Cited Literature

Hamilton, M.J., Davidson, A.D., Sibly, R.M., Brown, J.H., 2010. Universal scaling of production rates across mammalian lineages. Proceedings of the Royal Society B: Biological Sciences 278, 560–566.

Jones, K.E., Bielby, J., Cardillo, M., Fritz, S.A., O'Dell, J., Orme, C.D.L., Safi, K., Sechrest, W., Boakes, E.H., Carbone, C., Connolly, C., Cutts, M.J., Foster, J.K., Grenyer, R., Habib, M., Plaster, C.A., Price, S.A., Rigby, E.A., Rist, J., Teacher, A., Bininda-Emonds, O.R.P., Gittleman, J.L., Mace, G.M., Purvis, A., 2009. PanTHERIA: a species-level database of life history, ecology, and geography of extant and recently extinct mammals. Ecology 90, 2648–2648.

Luis, A.D., Hayman, D.T.S., O'Shea, T.J., Cryan, P.M., Gilbert, A.T., Pulliam, J.R.C., Mills, J.N., Timonin, M.E., Willis, C.K.R., Cunningham, A.A., Fooks, A.R., Rupprecht, C.E., Wood, J.L.N., Webb, C.T., 2013. A comparison of bats and rodents as reservoirs of zoonotic viruses: are bats special? Proc. R. Soc. B 280, 20122753.

Wilman, H., Belmaker, J., Simpson, J., de la Rosa, C., Rivadeneira, M.M., Jetz, W., 07/2014. EltonTraits 1.0: Species-level foraging attributes of the world's birds and mammals: Ecological Archives E095-178. Ecology 95, 2027–2027.
