# Supplementary material for: Prioritizing surveillance of Nipah virus in India
Source: PLoS Negl Trop Dis. 2019 Jun 27;13(6):e0007393. doi: 10.1371/journal.pntd.0007393 (PMC6597033; doi:10.1371/journal.pntd.0007393)
Supplement: S2 Table — Results of the first analysis (columns B and C) treat Nipah virus positivity as a binary response variable. Results of the second analysis (columns D and E) treat study effort as a Poisson distributed response variable (the number of citations returned from a search in Web of Science on the Latin binomials of each species). (PDF) [file pntd.0007393.s002.pdf]

S2 Table. The hyperparameter values and resulting trait profiles of two generalized boosted regression analyses on bats species from Asia, Australia, and Oceana

	batsNiVAsiaAUSOceana	WOS
**error structure**	bernoulli	poisson
**best.iter/n.trees**	44068/50000	17817/30000
**weights**	sum.sample.size	NA
**shrinkage**	0.0001	0.001
**interaction depth**	4	3
**min obs node**	4	2
**cv.folds**	10	10
**train/test**	AUC: 0.999/0.827	pseudo-R2: 0.996/**0.186**

var	rel.inf	var	rel.inf
aridity	9.638360727	RelAgeSexMat	11.65657231
X26.2_GR_MaxLat_dd	7.621557796	X28.1_Precip_Mean_mm	6.778379741
sppdens	7.529530543	X5.1_AdultBodyMass_g	6.418699114
X6.2_TrophicLevel	5.723405736	X13.1_AdultHeadBodyLen_mm	6.098737872
X13.1_AdultHeadBodyLen_mm	5.670099363	X10.1_PopulationGrpSize	5.995101247
X8.1_AdultForearmLen_mm	5.61236242	sppdens	5.012812569
X26.1_GR_Area_km2	5.050714137	torp	4.777556825
X30.1_AET_Mean_mm	4.540435213	aridity	4.48979419
spp.dens.per.km	3.521643034	Molossidae	4.296617033
X28.2_Temp_Mean_01degC	3.501174099	X27.4_HuPopDen_Change	4.177423581
X5.1_AdultBodyMass_g	3.443442414	X17.1_MaxLongevity_m	3.991020806
X25.1_WeaningAge_d	3.13136106	X26.2_GR_MaxLat_dd	3.750068438
X23.1_SexualMaturityAge_d	2.997032886	X30.1_AET_Mean_mm	3.675976085
X26.6_GR_MinLong_dd	2.622152484	X9.1_GestationLen_d	3.184935968
X15.1_LitterSize	2.526567837	Log10.mass_specific_production.	2.588402714
X26.5_GR_MaxLong_dd	2.390381043	X30.2_PET_Mean_mm	2.48734082
X10.1_PopulationGrpSize	2.28880821	X8.1_AdultForearmLen_mm	2.248176735
X26.4_GR_MidRangeLat_dd	2.272039279	postnatGR	1.708876314
X26.3_GR_MinLat_dd	2.058571479	X2.1_AgeatEyeOpening_d	1.693646589
X17.1_MaxLongevity_m	1.894555737	mig	1.643185933
X3.1_AgeatFirstBirth_d	1.626756227	BodyMass.Value	1.341545076
X28.1_Precip_Mean_mm	1.624749312	X13.2_NeonateHeadBodyLen_mm	1.21816856
X27.2_HuPopDen_Mean_n.km2	1.622108461	X26.5_GR_MaxLong_dd	1.157339721
X27.3_HuPopDen_5p_n.km2	1.591325153	X26.3_GR_MinLat_dd	1.066493911
X6.1_DietBreadth	1.243390219	X3.1_AgeatFirstBirth_d	1.049396863
X27.4_HuPopDen_Change	1.233329238	X26.6_GR_MinLong_dd	0.880297922
X30.2_PET_Mean_mm	1.016511048	X26.1_GR_Area_km2	0.77598315
mig	0.923204786	X25.1_WeaningAge_d	0.711653342
X16.1_LittersPerYear	0.892257496	X5.4_WeaningBodyMass_g	0.685405622
BodySizeRatio	0.705178872	X26.7_GR_MidRangeLong_dd	0.612717619
X26.7_GR_MidRangeLong_dd	0.648812584	AnnualBP	0.449184458
X5.3_NeonateBodyMass_g	0.470248593	X28.2_Temp_Mean_01degC	0.380511258
Hipposideridae	0.445415322	X26.4_GR_MidRangeLat_dd	0.348498206
X27.1_HuPopDen_Min_n.km2	0.351539463	X5.3_NeonateBodyMass_g	0.324856487
BodyMass.Value	0.330885752	X23.1_SexualMaturityAge_d	0.303228439
Rhinolophidae	0.294188504	BodySizeRatio	0.282611939
X9.1_GestationLen_d	0.240247293	X27.3_HuPopDen_5p_n.km2	0.2787099
RelAgeSexMat	0.142415191	X27.2_HuPopDen_Mean_n.km2	0.258680824
Diet.Inv	0.116951972	X27.1_HuPopDen_Min_n.km2	0.255468705
Log10.mass_specific_production.	0.102126033	X6.1_DietBreadth	0.224282926
Vespertilionidae	0.101272117	X15.1_LitterSize	0.220996227
torp	0.084576878	X18.1_BasalMetRate_mLO2hr	0.160559027
Diet.Fruit	0.064931942	X1.1_ActivityCycle	0.102648695
X5.4_WeaningBodyMass_g	0.031757703	spp.dens.per.km	0.102419966
postnatGR	0.024463266	X5.5_AdultBodyMass_g_EXT	0.057235817
X2.1_AgeatEyeOpening_d	0.016925238	X6.2_TrophicLevel	0.036529769
X18.1_BasalMetRate_mLO2hr	0.013088612	X16.1_LittersPerYear	0.018475701
AnnualBP	0.005848179	X5.2_BasalMetRateMass_g	0.017622639
Pteropodidae	0.000984523	Vespertilionidae	0.003322065
X13.2_NeonateHeadBodyLen_mm	0.000201887	Diet.Fruit	0.001339966
X5.2_BasalMetRateMass_g	0.000112646	Rhinolophidae	0.000396006
X1.1_ActivityCycle	0	Diet.Inv	8.06E-05
Molossidae	0	Hipposideridae	1.37E-05
		Pteropodidae	0
