# Supplementary material for: Prioritizing surveillance of Nipah virus in India
Source: PLoS Negl Trop Dis. 2019 Jun 27;13(6):e0007393. doi: 10.1371/journal.pntd.0007393 (PMC6597033; doi:10.1371/journal.pntd.0007393)
Supplement: S3 Table — A list of bat species in descending order NiV positivity as predicted by a generalized boosted regression model on species-level traits (pNiV+), and a binary column (NiV+) indicating seropositivity from all previously published work. (PDF) [file pntd.0007393.s003.pdf]

S3 Table. Species predictions

	p(NiV+)	NiV+
Pteropus_vampyrus	0.981519689	1
Rousettus_leschenaultii	0.98126489	1
Rousettus_amplexicaudatus	0.980860402	1
Cynopterus_sphinx	0.980633005	1
Cynopterus_brachyotis	0.972998413	1
Pteropus_hypomelanus	0.970118901	1
Hipposideros_armiger	0.968457483	1
Eonycteris_spelaea	0.968182258	1
Pteropus_giganteus	0.963824186	1
Rhinolophus_affinis	0.936616686	1
Eidolon_helvum	0.931401728	1
Pteropus_conspicillatus	0.880984142	1
Pteropus_lylei	0.866961871	1
Rousettus_aegyptiacus	0.857744052	0
Scotophilus_kuhlii	0.854011871	1
Taphozous_longimanus	0.851281755	0
Myotis_daubentonii	0.838289572	1
Hipposideros_pomona	0.83683462	1
Rhinolophus_sinicus	0.831939366	1
Myotis_ricketti	0.82621325	1
Taphozous_melanopogon	0.821532487	0
Rhinolophus_luctus	0.706128161	0
Chaerephon_plicatus	0.69840706	0
Macroglossus_minimus	0.680096375	0
Hipposideros_larvatus	0.673377598	1
Megaderma_spasma	0.61105439	0
Hipposideros_diadema	0.57938234	0
Saccolaimus_saccolaimus	0.511938362	0
Coelops_frithii	0.413812404	0
Hipposideros_ater	0.33447271	0
Hipposideros_pratti	0.313083967	0
Acerodon_jubatus	0.303890395	0
Plecotus_alpinus	0.29315513	0
Tylonycteris_robustula	0.279144839	0
Tylonycteris_pachypus	0.260467935	0
Mops_midas	0.245484662	0
Ptenochirus_jagori	0.240676298	0
Dobsonia_chapmani	0.239484031	0
Myotis_nipalensis	0.232722021	0
Pipistrellus_tenuis	0.222638515	0
Hypsugo_alaschanicus	0.216269464	0
Hipposideros_cineraceus	0.210988692	0
Pteropus_tonganus	0.208445065	0
Kerivoula_hardwickii	0.200440046	0

Harpyionycteris_whiteheadi	0.199531779	0
Taphozous_theobaldi	0.198234681	0
Myotis_frater	0.184551066	0
Myotis_formosus	0.18234968	0
Pteropus_poliocephalus	0.176634282	0
Rhinolophus_clivosus	0.175416447	0
Myotis_muricola	0.163772409	0
Myotis_adversus	0.163006441	0
Scotophilus_heathii	0.162605957	0
Triaenops_persicus	0.161768975	0
Pteralopex_acrodonta	0.160945512	0
Hipposideros_galeritus	0.158362323	0
Macroglossus_sobrinus	0.15311241	0
Hypsugo_savii	0.150116139	0
Rhinopoma_hardwickii	0.148949132	0
Pteropus_scapulatus	0.148484699	0
Murina_cyclotis	0.147290462	0
Kerivoula_picta	0.14399335	0
Rhinolophus_ferrumequinum	0.143561542	0
Lasiurus_cinereus	0.142127577	0
Hipposideros_lylei	0.140520208	0
Pipistrellus_javanicus	0.139780782	0
Pipistrellus_pygmaeus	0.139625499	0
Scotophilus_collinus	0.13956449	0
Megaderma_lyra	0.139074057	0
Aproteles_bulmerae	0.136958513	0
Harpiocephalus_harpia	0.135881207	0
Myotis_montivagus	0.135111264	0
Chaerephon_pumilus	0.134950479	0
Rhinolophus_siamensis	0.134119877	0
Ia_io	0.132331777	0
Syconycteris_australis	0.132244001	0
Dobsonia_moluccensis	0.132181921	0
Rhinolophus_pearsonii	0.12997704	0
Kerivoula_lenis	0.129659293	0
Mops_sarasinorum	0.129354203	0
Taphozous_perforatus	0.127974891	0
Myotis_australis	0.127119741	0
Nyctalus_plancyi	0.124740201	0
Paranyctimene_tenax	0.124296708	0
Pteropus_neohibernicus	0.124037413	1
Pteropus_alecto	0.121689014	0
Rousettus_linduensis	0.117176156	0
Pteropus_loochoensis	0.117015545	0
Hipposideros_scutinares	0.11675507	0
Eptesicus_nilssonii	0.116589955	0
Pipistrellus_ceylonicus	0.113728783	0
Myotis_mystacinus	0.113364457	0

Pteropus_pumilus	0.113204302	0
Hipposideros_bicolor	0.112883081	0
Hypsugo_cadornae	0.111827805	0
Myotis_davidii	0.110728487	0
Plecotus_kolombatovici	0.110687111	0
Myotis_bucharensis	0.110517553	0
Nyctimene_rabori	0.1085244	0
Pteropus_mariannus	0.107242159	0
Pteropus_rayneri	0.103588573	0
Tadarida_aegyptiaca	0.101377471	0
Nyctalus_noctula	0.099703065	0
Rhinolophus_yunanensis	0.099306847	0
Pipistrellus_pipistrellus	0.099288213	0
Taphozous_nudiventris	0.097981952	0
Myotis_myotis	0.09603143	0
Hypsugo_anthonyi	0.095217617	0
Hypsugo_pulveratus	0.0945764	0
Vespertilio_murinus	0.092886309	0
Murina_grisea	0.092460631	0
Hypsugo_joffrei	0.091537434	0
Rhinolophus_macrotis	0.091334816	0
Pipistrellus_abramus	0.091089292	0
Scotomanes_ornatus	0.090457357	0
Hipposideros_lankadiva	0.089907806	0
Myotis_hasseltii	0.086122708	0
Tadarida_teniotis	0.085712526	0
Megaerops_ecaudatus	0.085473077	0
Hipposideros_caffer	0.083115308	0
Pipistrellus_nathusii	0.082818136	0
Myotis_ater	0.082679725	0
Eptesicus_serotinus	0.081057688	0
Chaerephon_jobensis	0.080788784	0
Barbastella_barbastellus	0.080407894	0
Myotis_laniger	0.079728126	0
Haplonycteris_fischeri	0.079139153	0
Barbastella_leucomelas	0.078302096	0
Hipposideros_cervinus	0.078275982	0
Pteropus_intermedius	0.075976303	0
Myotis_blythii	0.075689574	0
Myotis_horsfieldii	0.075663222	0
Plecotus_auritus	0.075188249	0
Hypsugo_imbricatus	0.074870421	0
Falsistrellus_affinis	0.074203231	0
Myotis_moluccarum	0.074135364	0
Nycteris_thebaica	0.07278739	0
Myotis_chinensis	0.071146803	0
Myotis_dasycneme	0.070005126	0
Myotis_siligorensis	0.069147223	0

Myotis_bocagii	0.068563819	0
Hypsugo_vordermanni	0.068502517	0
Chaerephon_solomonis	0.067335172	0
Pteropus_melanotus	0.066233236	0
Hipposideros_turpis	0.066131934	0
Chaerephon_bregullae	0.065596738	0
Pteropus_ornatus	0.065176353	0
Philetor_brachypterus	0.064047172	0
Chaerephon_johorensis	0.063046588	0
Pipistrellus_kuhlii	0.062800707	0
Dyacopterus_spadiceus	0.062527917	0
Rhinolophus_acuminatus	0.061466461	0
Pteralopex_anceps	0.059349294	0
Nyctimene_albiventer	0.059271659	0
Rhinolophus_megaphyllus	0.058667623	0
Vespertilio_sinensis	0.058265971	0
Asellia_tridens	0.057735738	0
Glischropus_tylopus	0.057630268	0
Rhinolophus_philippinensis	0.057363745	0
Hypsugo_kitcheneri	0.057074836	0
Otomops_wroughtoni	0.056867021	0
Hipposideros_halophyllus	0.056547479	0
Rhinolophus_hipposideros	0.055568036	0
Hypsugo_macrotis	0.0554381	0
Pteropus_samoensis	0.053792603	0
Mops_mops	0.053614564	0
Pteropus_dasymallus	0.053499636	0
Pteropus_macrotis	0.053319036	0
Myotis_fimbriatus	0.053317388	0
Cynopterus_horsfieldii	0.053228566	0
Sphaerias_blanfordi	0.053075791	0
Hipposideros_lekaguli	0.052753045	0
Latidens_salimalii	0.052312271	0
Pipistrellus_rueppellii	0.051959341	0
Kerivoula_papillosa	0.051632106	0
Arielulus_circumdatus	0.051242064	0
Cynopterus_titthaecheilus	0.049815594	0
Rhinolophus_stheno	0.049156984	0
Hypsugo_ariel	0.048982168	0
Pteropus_leucopterus	0.048769422	0
Eptesicus_gobiensis	0.048608243	0
Rhinolophus_shortridgei	0.048359272	0
Dobsonia_minor	0.048263119	0
Pipistrellus_paterculus	0.04825723	0
Rhinopoma_microphyllum	0.0481593	0
Hypsugo_arabicus	0.047830715	0
Rhinolophus_trifoliatus	0.046851355	0
Hipposideros_speoris	0.046442153	0

Rhinolophus_blasii	0.04586363	0
Rhinonicteris_aurantia	0.045810143	0
Hypsugo_lophurus	0.045241958	0
Rhinolophus_shameli	0.045237849	0
Pteropus_melanopogon	0.044955757	0
Rhinolophus_arcuatus	0.044325944	0
Saccolaimus_flaviventris	0.043685944	0
Pteropus_chrysoproctus	0.043643099	0
Rhinolophus_paradoxolophus	0.043398014	0
Rhinolophus_marshalli	0.042979336	0
Pteropus_molossinus	0.042886667	0
Rousettus_spinalatus	0.042866788	0
Dobsonia_praedatrix	0.041670786	0
Megaerops_niphanae	0.041342339	0
Otonycteris_hemprichii	0.04126953	0
Pteropus_speciosus	0.040368875	0
Rhinolophus_coelophyllus	0.039948149	0
Rhinolophus_thomasi	0.039841396	0
Phoniscus_jagorii	0.039830144	0
Tadarida_insignis	0.039438321	0
Rhinolophus_lepidus	0.038925644	0
Cynopterus_minutus	0.037764186	0
Murina_ussuriensis	0.037533441	0
Cheiromeles_torquatus	0.037307823	0
Murina_hilgendorfi	0.037306282	0
Myotis_csorbai	0.036847852	0
Acerodon_celebensis	0.036717829	0
Paranyctimene_raptor	0.035579052	0
Penthetor_lucasi	0.03493049	0
Rhinolophus_malayanus	0.034579694	0
Aselliscus_stoliczkanus	0.034355264	0
Myotis_nattereri	0.033987299	0
Nyctimene_major	0.033855751	0
Nyctalus_leisleri	0.033492392	0
Rhinolophus_pusillus	0.033216751	0
Eonycteris_major	0.032864492	0
Emballonura_alecto	0.032838924	0
Emballonura_semicaudata	0.032817069	0
Nyctalus_lasiopterus	0.032734425	0
Chironax_melanocephalus	0.032587606	0
Pipistrellus_coromandra	0.032526981	0
Eptesicus_japonensis	0.032514644	0
Rhinolophus_convexus	0.03239963	0
Nyctalus_aviator	0.032065222	0
Dobsonia_peronii	0.032012978	0
Rhinolophus_rex	0.031791777	0
Taphozous_georgianus	0.031744541	0
Myotis_bechsteinii	0.031695515	0

Nyctophilus_geoffroyi	0.031560767	0
Myotis_brandtii	0.031509586	0
Rousettus_celebensis	0.031335024	0
Eonycteris_robusta	0.031257663	0
Nyctimene_robinsoni	0.030392767	0
Nyctimene_aello	0.030245729	0
Macroderma_gigas	0.030224047	0
Dobsonia_exoleta	0.030150329	0
Hesperoptenus_tickelli	0.030081524	0
Rhinolophus_madurensis	0.030060445	0
Myotis_altarium	0.029809458	0
Rhinolophus_borneensis	0.029559896	0
Eptesicus_dimissus	0.029423279	0
Emballonura_monticola	0.029229625	0
Rhinolophus_osgoodi	0.029144476	0
Dobsonia_inermis	0.029011943	0
Chalinolobus_gouldii	0.028696773	0
Acerodon_mackloti	0.02823819	0
Mormopterus_beccarii	0.028154257	0
Pteropus_faunulus	0.028131945	0
Nyctophilus_gouldi	0.027886506	0
Tadarida_latouchei	0.027846885	0
Nyctalus_furvus	0.027771519	0
Hipposideros_grandis	0.027670317	0
Mystacina_tuberculata	0.027547134	0
Harpyionycteris_celebensis	0.027469507	0
Rhinolophus_rouxii	0.027411536	0
Rhinolophus_euryale	0.027390898	0
Craseonycteris_thonglongyai	0.027244913	0
Melonycteris_woodfordi	0.027071507	0
Mormopterus_planiceps	0.026646006	0
Chalinolobus_tuberculatus	0.026556607	0
Hipposideros_ridleyi	0.026532314	0
Vespadelus_pumilus	0.026489146	0
Ptenochirus_minor	0.026414208	0
Pteralopex_atrata	0.026274985	0
Styloctenium_wallacei	0.026187001	0
Arielulus_aureocollaris	0.026140592	0
Vespadelus_finlaysoni	0.026139111	0
Thoopterus_nigrescens	0.026106196	0
Murina_huttoni	0.026039822	0
Rhinolophus_beddomei	0.02602745	0
Rhinolophus_rufus	0.025915404	0
Cheiromeles_parvidens	0.025622575	0
Hipposideros_nequam	0.025563265	0
Myotis_annectans	0.025411561	0
Pteropus_griseus	0.025340157	0
Myotis_rosseti	0.024969959	0

Rhinolophus_bocharicus	0.024812432	0
Melonycteris_melanops	0.02448037	0
Pteropus_ualanus	0.024381097	0
Rhinolophus_euryotis	0.024316786	0
Nyctimene_cephalotes	0.024259989	0
Hipposideros_edwardshilli	0.024130375	0
Plecotus_austriacus	0.024098449	0
Nyctimene_draconilla	0.023992689	0
Hipposideros_rotalis	0.023862162	0
Dobsonia_viridis	0.023817819	0
Pteropus_pelewensis	0.023798808	0
Myotis_emarginatus	0.023777803	0
Eptesicus_pachyotis	0.023549724	0
Hipposideros_inornatus	0.023441991	0
Murina_tubinaris	0.023328412	0
Mormopterus_doriae	0.02331353	0
Nyctalus_montanus	0.02321472	0
Pteropus_mahaganus	0.023179001	0
Vespadelus_caurinus	0.023161806	0
Nyctophilus_bifax	0.023072569	0
Pteropus_nitendiensis	0.02286701	0
Myotis_hajastanicus	0.022852959	0
Murina_leucogaster	0.022787192	0
Pteropus_fundatus	0.022739874	0
Hipposideros_fulvus	0.02267089	0
Scotorepens_balstoni	0.022647026	0
Myotis_hermani	0.022578145	0
Rhinolophus_subbadius	0.022465929	0
Murina_aurata	0.02239392	0
Tadarida_australis	0.022330223	0
Falsistrellus_petersi	0.022286877	0
Otopteropus_cartilagonodus	0.022270917	0
Myotis_yanbarensis	0.022200943	0
Murina_ryukyuana	0.022108803	0
Myotis_annamiticus	0.022021219	0
Cynopterus_luzoniensis	0.021926195	0
Hipposideros_dinops	0.021780698	0
Taphozous_australis	0.021682676	0
Balionycteris_maculata	0.021633245	0
Rhinolophus_inops	0.021482851	0
Murina_florium	0.021295692	0
Notopteris_macdonaldi	0.021287557	0
Myotis_capaccinii	0.021178473	0
Myotis_bombinus	0.021109073	0
Pteropus_admiralitatum	0.021084527	0
Dobsonia_beauforti	0.021063001	0
Nyctophilus_timoriensis	0.020999643	0
Murina_tenebrosa	0.020858077	0

Dobsonia_pannietensis	0.02078933	0
Dobsonia_anderseni	0.020661523	0
Alionycteris_paucidentata	0.02061043	0
Pipistrellus_stenopterus	0.020553779	0
Rhinolophus_subrufus	0.020521994	0
Rhinolophus_mehelyi	0.020521991	0
Pteropus_caniceps	0.020503808	0
Myotis_ikonnikovi	0.020388806	0
Hipposideros_breviceps	0.020338327	0
Hipposideros_inexpectatus	0.020324535	0
Hipposideros_semoni	0.020315793	0
Neopteryx_frosti	0.020263702	0
Pipistrellus_papuanus	0.020083852	0
Taphozous_kapalgensis	0.019966728	0
Hipposideros_megalotis	0.0199025	0
Hipposideros_hypophyllus	0.019834638	0
Hipposideros_madurae	0.0197771	0
Myotis_macropus	0.01974131	0
Syconycteris_hobbit	0.019656006	0
Myotis_pruinosus	0.01961519	0
Hipposideros_pygmaeus	0.019525743	0
Pipistrellus_endoi	0.019475648	0
Pipistrellus_adamsi	0.019406265	0
Acerodon_humilis	0.019400878	0
Myotis_pequinius	0.019383163	0
Mosia_nigrescens	0.019358307	0
Aselliscus_tricuspidatus	0.019343975	0
Saccolaimus_mixtus	0.019055429	0
Murina_fusca	0.01903134	0
Rhinolophus_formosae	0.019019786	0
Hipposideros_sorenseni	0.018958386	0
Hipposideros_orbiculus	0.018903498	0
Rhinolophus_mitratus	0.018856061	0
Hipposideros_durgadasi	0.018525408	0
Pipistrellus_wattsi	0.018284883	0
Rousettus_bidens	0.018192834	0
Vespadelus_douglasorum	0.018130002	0
Pipistrellus_westralis	0.018100685	0
Rhinolophus_celebensis	0.018061788	0
Scotophilus_celebensis	0.017997349	0
Rhinolophus_cognatus	0.017840793	0
Myotis_insularum	0.017753467	0
Hipposideros_obscurus	0.017728609	0
Myotis_gomantongensis	0.017632346	0
Eptesicus_bottae	0.017533154	0
Hipposideros_stenotis	0.017530372	0
Nycteris_tragata	0.017429714	0
Kerivoula_whiteheadi	0.017389092	0

Arielulus_cuprosus	0.017341482	0
Nyctimene_certans	0.017339498	0
Scotozous_dormeri	0.017324696	0
Pteropus_tuberculatus	0.017258676	0
Pipistrellus_angulatus	0.017194429	0
Nyctimene_sanctacrucis	0.017072621	0
Pteropus_gilliardorum	0.016972538	0
Tadarida_kuboriensis	0.016939793	0
Vespadelus_troughtoni	0.01691859	0
Pteropus_personatus	0.016613662	0
Megaerops_wetmorei	0.016543845	0
Pteropus_rennelli	0.016519071	0
Scotoecus_pallidus	0.01651262	0
Aethalops_alecto	0.016480242	0
Nyctophilus_heran	0.016392087	0
Chalinolobus_morio	0.01637916	0
Dobsonia_crenulata	0.0163722	0
Myotis_macrodactylus	0.016369424	0
Pteropus_ocularis	0.016345892	0
Myotis_macrotarsus	0.016343719	0
Rhinolophus_nereis	0.01631629	0
Eudiscopus_denticulus	0.016277022	0
Chalinolobus_dwyeri	0.016272847	0
Pteropus_keyensis	0.016157561	0
Myotis_schaubi	0.016152223	0
Nyctimene_keasti	0.016146527	0
Pteralopex_taki	0.016129291	0
Pteropus_aruensis	0.016128159	0
Myotis_longipes	0.015973843	0
Nyctophilus_arnhemensis	0.015936085	0
Nyctophilus_walkeri	0.015914761	0
Hipposideros_doriae	0.015913264	0
Plecotus_taivanus	0.015894118	0
Arielulus_torquatus	0.015886314	0
Notopteris_neocaledonica	0.015881736	0
Pteropus_vetulus	0.015840362	0
Hipposideros_maggietaylorae	0.01578409	0
Myotis_stalkeri	0.015778182	0
Nyctimene_malaitensis	0.015771287	0
Scotorepens_orion	0.015754119	0
Pteropus_anetianus	0.015677312	0
Otomops_johnstonei	0.015673012	0
Pteropus_woodfordi	0.015580941	0
Nyctimene_cyclotis	0.015527382	0
Otomops_formosus	0.015518668	0
Pteropus_pohlei	0.015518369	0
Hipposideros_dyacorum	0.015472805	0
Coelops_robinsoni	0.015445111	0

Pipistrellus_collinus	0.015412055	0
Eptesicus_tatei	0.015394857	0
Phoniscus_papuensis	0.01535264	0
Rhinolophus_robinsoni	0.015282935	0
Nyctimene_vizcaccia	0.015178731	0
Vespadelus_regulus	0.015127122	0
Syconycteris_carolinae	0.015076335	0
Myotis_oreias	0.015012078	0
Anthops_ornatus	0.014907652	0
Chalinolobus_picatus	0.014853811	0
Rhinolophus_canuti	0.014809329	0
Kerivoula_flora	0.014795983	0
Scotorepens_sanborni	0.014748479	0
Dyacopterus_brooksi	0.014712782	0
Eptesicus_bobrinskoi	0.014677398	0
Otomops_secundus	0.014650114	0
Hipposideros_coronatus	0.014647665	0
Nyctimene_minutus	0.014635579	0
Kerivoula_pellucida	0.014563726	0
Hipposideros_papua	0.014525438	0
Myotis_sicarius	0.014463256	0
Nyctimene_masalai	0.014462639	0
Scotorepens_greyii	0.01439802	0
Falsistrellus_mordax	0.014351469	0
Rhinolophus_virgo	0.014217773	0
Dobsonia_emersa	0.014199318	0
Nycteris_javanica	0.014184092	0
Falsistrellus_tasmaniensis	0.014041014	0
Hesperoptenus_blanfordi	0.013939568	0
Taphozous_hilli	0.013847971	0
Scoteanax_rueppellii	0.013843769	0
Hipposideros_macrobullatus	0.013735889	0
Asellia_patrizii	0.013721163	0
Vespadelus_darlingtoni	0.013677668	0
Phoniscus_atrox	0.013628961	0
Vespadelus_baverstocki	0.013463326	0
Taphozous_troughtoni	0.013431678	0
Rhinolophus_keyensis	0.013314903	0
Pteropus_lombocensis	0.013275904	0
Arielulus_societatis	0.013264892	0
Kerivoula_minuta	0.013259595	0
Emballonura_furax	0.013170298	0
Hesperoptenus_tomesi	0.0131615	0
Otomops_papuensis	0.013043549	0
Megaerops_kusnotoi	0.012978965	0
Hipposideros_muscinus	0.012880017	0
Hipposideros_wollastoni	0.012819456	0
Vespadelus_vulturnus	0.012808788	0

Pteralopex_pulchra	0.012798144	0
Falsistrellus_mackenziei	0.012798082	0
Mormopterus_loriae	0.012747637	0
Kerivoula_intermedia	0.012661649	0
Hipposideros_calcaratus	0.012617826	0
Hipposideros_corynophyllus	0.012615216	0
Rhinopoma_muscatellum	0.01260622	0
Rhinolophus_montanus	0.01259774	0
Emballonura_raffrayana	0.01247655	0
Pharotis_imogene	0.012473248	0
Murina_suilla	0.012234073	0
Glischropus_javanus	0.012200787	0
Hipposideros_sumbae	0.012175732	0
Chalinolobus_nigrogriseus	0.012105404	0
Acerodon_leucotis	0.012090745	0
Hipposideros_coxi	0.012053298	0
Emballonura_dianae	0.011971618	0
Hipposideros_pelingensis	0.011953446	0
Hesperoptenus_gaskelli	0.011951185	0
Pteropus_capistratus	0.011923431	0
Murina_puta	0.011852261	0
Kerivoula_muscina	0.011778683	0
Cynopterus_nusatenggara	0.011739109	0
Eptesicus_nasutus	0.011636732	0
Murina_rozendaali	0.011599396	0
Pteropus_temminckii	0.011594804	0
Nyctophilus_microtis	0.011539434	0
Myotis_ridleyi	0.011501145	0
Pteropus_cognatus	0.011394246	0
Nyctophilus_nebulosus	0.011381498	0
Chalinolobus_neocaledonicus	0.01137862	0
Murina_aenea	0.011305842	0
Hipposideros_demissus	0.011292283	0
Nyctophilus_microdon	0.011133255	0
Taphozous_achates	0.01112003	0
Melonycteris_fardoulisi	0.01111037	0
Emballonura_serii	0.01108177	0
Mormopterus_norfolkensis	0.011062158	0
Hesperoptenus_doriae	0.01104153	0
Aethalops_aequalis	0.010974066	0
Emballonura_beccarii	0.01092517	0
Rhinolophus_sedulus	0.010735938	0
Hipposideros_crumeniferus	0.010610038	0
Rhinolophus_creaghi	0.010475879	0
Pipistrellus_minahassae	0.009861061	0
Kerivoula_myrella	0.009601051	0
Kerivoula_agnella	0.009523992	0
