# Supplementary figures and images for: Prioritizing surveillance of Nipah virus in India
Source: PLoS Negl Trop Dis. 2019 Jun 27;13(6):e0007393. doi: 10.1371/journal.pntd.0007393 (PMC6597033; doi:10.1371/journal.pntd.0007393)

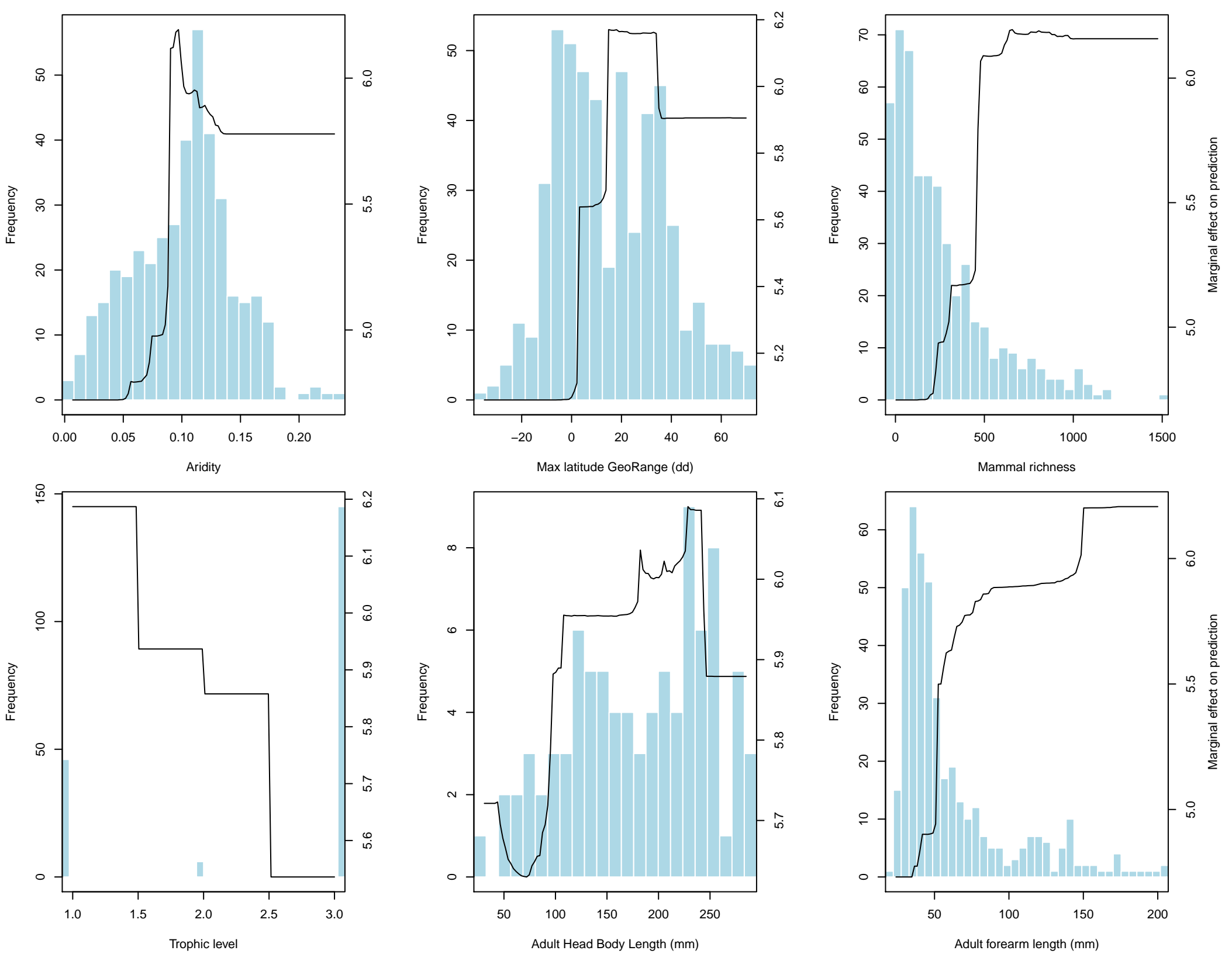

Supplement: S1 Fig — The frequency histogram of trait value distribution across all bat species (blue bars) is overlaid with a black line indicating model sensitivity to trait values for classification accuracy. (PDF) [file pntd.0007393.s004.pdf]
