# Supplementary material for: Prioritizing surveillance of Nipah virus in India
Source: PLoS Negl Trop Dis. 2019 Jun 27;13(6):e0007393. doi: 10.1371/journal.pntd.0007393 (PMC6597033; doi:10.1371/journal.pntd.0007393)
Supplement: S2 Checklist — Table of included papers. (PDF) [file pntd.0007393.s006.pdf]

included studies

	studyid
1	Wacharapluesadee A Longitudinal Study of the Prevalence of Nipah Virus in Pteropus lylei Bats in Thailand: Evidence for Seasonal Preference in Disease Transmission
62	Kashiwazaki A solid-phase blocking ELISA for detection of antibodies to Nipah virus
64	Pulliam Agricultural intensification, priming for persistence and the emergence of Nipah virus: a lethal bat-borne zoonosis
66	Li Antibodies to Nipah or Nipah-like Viruses in Bats , China
125	Wacharapluesadee Bat Nipah Virus, Thailand
136	Peel Continent-wide panmixia of an African fruit bat facilitates transmission of potentially zoonotic viruses
151	Hayman Evidence of Henipavirus Infection in West African Fruit Bats
157	Epstein Feral Cats and Risk for Nipah Virus Transmission
159	Lehlé Henipavirus and Tioman Virus Antibodies in Pteropodid Bats, Madagascar
162	Sendow Henipavirus in Pteropus vampyrus Bats, Indonesia
165	Epstein Henipavirus Infection in Fruit Bats (Pteropus gigangeus), India
171	Peel Henipavirus Neutralising Antibodies in an Isolated Island Population of African Fruit Bats
172	Chua Isolation of Nipah virus from Malaysian Island flying-foxes
173	Wacharapluesadee Molecular characterization of Nipah virus from Pteropus hypomelanus in Southern Thailand
180	Hsu Nipah Virus Encephalitis Reemergence, Bangladesh
181	Reynes Nipah Virus in Lyle's Flying Foxes , Cambodia
196	Sendow Nipah Virus in the Fruit Bat Pteropus vampyrus in Sumatera , Indonesia
200	Johara Nipah Virus Infection in Bats (Order Chiroptera) in Peninsular Malaysia
214	Shirai Nipah Virus Survey of Flying Foxes in Malaysia
221	Breed Prevalence of Henipavirus and Rubulavirus Antibodies in Pteropid Bats, Papau New Guinea
222	Rahman Risk Factors for Nipah Virus Infection among Pteropid Bats , Peninsular Malaysia
251	Sendow Screening for Nipah Virus Infection in West Kalimantan Province , Indonesia
255	Hasebe Serologic Evidence of Nipah Virus Infection in Bats , Vietnam
259	Yadav Short Report : Detection of Nipah Virus RNA in Fruit Bat (Pteropus giganteus) from India
265	Breed The Distribution of Henipaviruses in Southeast Asia and Australasia: Is Wallace's Line a Barrier to Nipah Virus?
